# Chemistry Teachers’ Perception of Students’ Difficulties in Reading and Drawing Chemical Structures

**DOI:** 10.1021/acs.jchemed.5c00204

**Published:** 2026-02-24

**Authors:** Lars-Jochen Thoms, Gina Blick, Lukas Schmidt, Florian Furrer, Mitra Purandare, Frieder Loch, Johannes Huwer

**Affiliations:** † Chair of Science Education, 87678 Thurgau University of Teacher Education, Kreuzlingen 8280, Switzerland; ‡ Chair of Science Education, Department of Chemistry, 26567 University of Konstanz, Konstanz 78464, Germany; ¶ Thurgau University of Teacher Education, Kreuzlingen 8280, Switzerland; § Department of Computer Science, 112888 Eastern Switzerland University of Applied Sciences, Rapperswil 8640, Switzerland

**Keywords:** Chemical Education Research, High School/First Year/Second Year Undergraduate, Visualization in Chemistry, Structural Formula Errors, Misconceptions/Conceptual Understanding, Chemical Structure Representations, Representational Competence, Lewis Structures and Skeletal Formulas

## Abstract

The acquisition of fundamental knowledge in chemical formula language and the ability to graphically represent chemical structures are critical components of chemistry education. Various structural representational forms, such as molecular formulas, Lewis formulas, and skeletal formulas, serve different purposes in describing chemical compounds and require specialized skills to interpret and create. While experts in the field can seamlessly transition between these representational forms, students often face challenges in building the necessary mental models and understanding the design conventions. This study investigates how chemistry teachers perceive and address students’ difficulties in reading and drawing chemical structural formulas. Through a survey of 116 chemistry teachers from Switzerland, Germany, Austria, and Denmark, the study explores the introduction of structural representational forms in classrooms, common student errors, and strategies employed to mitigate these errors. Teachers frequently use simpler chemical compounds, such as alkanes and alcohols, to introduce various representational forms and to diagnose typical errors. The most common errors identified include missing or excess atoms, incorrect spatial orientation, and violation of the octet rule. Findings emphasize the importance of gradually introducing complexity in chemical notations, starting with basic structures and progressively advancing to more complex spatial representational forms. Teachers highlight the value of repetition, targeted exercises, and interactive tools, such as augmented reality, to enhance students’ spatial reasoning and conceptual understanding. These insights underscore the need for innovative educational resources to support individualized learning paths and improve proficiency in chemical representation.

## Graphical abstract

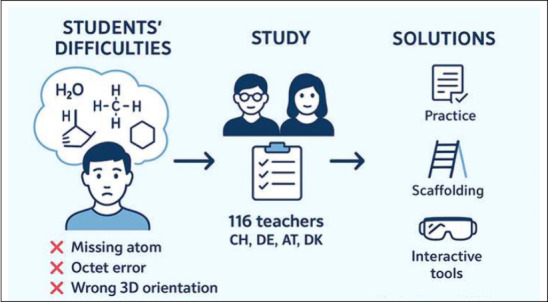

## Introduction

For the description of chemical compounds and reaction mechanisms, a large number of abstract formalisms have historically evolved in the discipline of chemistry, especially in the field of organic chemistry.^1^ These include, among others, molecular formulas, condensed formulas, Kekulé and Lewis formulas, skeletal formulas, and projections, like Newman, Sawhorse, Fischer, and Haworth projections (Figure 1). Each of these representational forms was developed to emphasize different structural or spatial aspects of molecules and thus serve specific communicative and cognitive functions in the practice of chemistry.^2,3^

**Figure 1:**
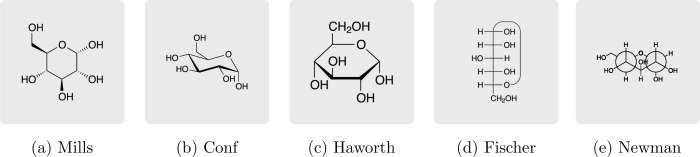
Projections of α-d-glucopyranose: (a) Mills projection (Mills), (b) skeletal formula in chair conformation (Conf), (c) Haworth projection (Haworth), (d) Fischer projection (Fischer), (e) Newman projection (Newman).

In this article, we distinguish representational forms (classes of external representations defined by common design conventions) from representations (specific instantiations for a given compound and task). We use the term *formula* exclusively for 2D symbolic notations with or without spatial information (e.g., molecular, condensed/semistructural, Lewis, valence-line/line-bond, skeletal, and wedge–dash formulas) and reserve *model* for submicroscopic 3D depictions (for a glossary and a hierarchical view, see Supporting Information (SI), Section 1, Table S1 and Figure S1).

Expert chemists select an appropriate representational form depending on the application purpose and context but are also able to translate between different representational forms as needed.^3–11^ This representational competence is not innate but rather developed through education and practice.^3,12,13^ Because expert communication of chemical knowledge depends on the use of appropriate representations,^5,6,9,12^ chemistry students are introduced to various representational forms early and gradually develop the representational fluency needed to participate in disciplinary discourse.^9,14^ In particular, structural formulas serve as a central representational form that experts use within the chemical community to construct, justify, and communicate claims.^6,12^ Accordingly, national standards and curricula typically emphasize the ability to express chemical relationships using appropriate representational forms as a core competency (for a curricular analysis, see SI, Section 2).

However, the degree of specificity with which structural representational forms are named in curricula varies widely across countries and, within federal systems, across states or cantons. In most cases, representational forms are required only in generic terms, with explicit forms cited infrequently (see SI, Table S2). Consequently, standards and curricula only weakly constrain the representational forms that are ultimately enacted in classrooms.

The historical emergence of domain-specific chemical representational forms—particularly in organic chemistry—provides important context for understanding their curricular relevance as tools for thinking, communication, and scientific reasoning.^1^ Chemistry, and organic chemistry in particular, has developed a wide range of abstract representational forms to address the complexity of invisible molecular structures and transformations. These representational forms are deeply rooted in the history of the discipline, reflecting both conceptual innovation and practical necessity. Their continued presence in curricula mirrors the diverse reasoning and visualization strategies required to understand, explain, and manipulate chemical phenomena. In the 19th century, organic chemistry faced a dramatic increase in the number and complexity of known compounds.^15–17^ To manage this complexity, chemists developed increasingly abstract and systematized representational systems.^18^ This development included the transition from Berzelian symbols to so-called “line-and-letter” formulas, shaped not only by emerging theoretical frameworks—such as structure theory and valence concepts—but also by practical constraints in printing, teaching, and international scientific communication.^19^ The proliferation of representational forms was thus not merely a stylistic choice but a response to diverse epistemic and pedagogical demands. Chemists sought precise visual tools to convey both the connectivity and spatial arrangement of atoms in molecules. Over time, particular representational forms have become preferred conventions within subfields of chemistry, aligning with—and shaping—how those communities communicate and reason:^6,12,19^ Lewis formulas are used in general and inorganic chemistry to depict valence electrons and bonding patterns; Kekulé and skeletal formulas are foundational in organic chemistry for illustrating molecular frameworks; Fischer and Haworth projections are central in carbohydrate chemistry; Newman projections support conformational analysis; and wedge–dash formulas are crucial for representing stereochemistry. Each form highlights different structural or reactive aspects of molecules, functioning as a visual language tailored to specific contexts of explanation, prediction, or argumentation.^18,20^ Accordingly, organic chemistry curricula introduce a variety of representational forms not for redundancy, but to cultivate students’ fluency in navigating multiple dimensions of molecular understanding—from connectivity and conformation to reactivity and spatial orientation. Mastery of these systems is essential for engaging in disciplinary discourse and for developing the kind of expert reasoning that underpins advanced chemical thinking.

The International Union of Pure and Applied Chemistry (IUPAC) provides recommendations and guidelines for the design of structural formulas,^21,22^ and dedicated textbooks for structural notations in organic chemistry are available.^23^ In particular, IUPAC names and describes the following notations: *Lewis formula*, *Line formula* and *Kekulé structure*. Additionally, IUPAC gives comprehensive recommendations on how to design structural formulas.^21,22^

IUPAC describes the Lewis formula (also: electron dot or Lewis structure) as “molecular structure in which the valency electrons are shown as dots so placed between the bonded atoms that one pair of dots represents two electrons or one covalent (single) bond [...]. A double bond is represented by two pairs of dots, etc.”^24^ Only as a last sentence is a note added in brackets: “Bonding pairs of electrons are usually denoted by lines, representing covalent bonds, as in line formulas.”^24^ This means that the line formula described as follows is not clearly differentiated from the previous Lewis formula: “A two-dimensional representation of molecular entities in which atoms are shown joined by lines representing single or multiple bonds, without any indication or implication concerning the spatial direction of bonds.”^24^ Furthermore, a skeletal structure is described as a “sequence of atoms in the constitutional unit(s) of a macromolecule, an oligomer molecule, a block or a chain which defines the essential topological representation.”^24^ A Kekulé structure (for aromatic compounds) is defined as a “representation of an aromatic molecular entity (such as benzene), with fixed alternating single and double bonds, in which interactions between multiple bonds are assumed to be absent.”^24^

However, it should not be underestimated how versatile and complex the chemical formula language is. The representational forms shown in Figure 2 were taken from different textbooks and molecular modeling software (for references, see SI, Table S3) and describe, as needed, the composition (Figure 2a), the structure of the compound shown (Figure 2b–2f), and give indications of the spatial arrangement (Figure 2g,2h). To clearly distinguish between the representational forms assessed in this article, we will use the nomenclature described in the SI (see Section 1) below.

**Figure 2:**
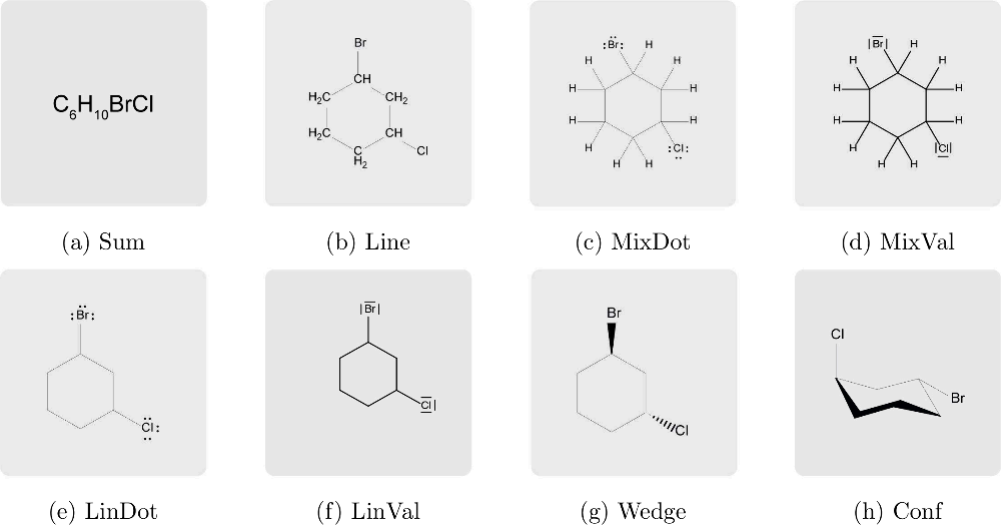
Exemplary representations of (1*R*,3*R*)-1-bromo-3-chlorocyclohexane assessed in this study: (a) molecular formula (Sum), (b) condensed structural formula (Line), (c) mixed line formula with lone pairs in dot notation and explicit hydrogens (MixDot), (d) mixed line formula with lone pairs in valence-line notation and explicit hydrogens (MixVal), (e) line formula with lone pairs in dot notation and implicit hydrogens (LinDot), (f) line formula with lone pairs in valence-line notation and implicit hydrogens (LinVal), (g) wedge–dash notation with implicit lone pairs and implicit hydrogens (Wedge), (h) skeletal formula in chair conformation (Conf).

In systems with binding state/cantonal standards and internal school syllabi (e.g., Switzerland, Germany, and the USA), curricula provide the legal framework for instruction.^25^ Our present study focuses on the enacted level—teachers’ professional expectations and reported practice regarding specific representational forms (for a conceptual framework, see SI, section 4). Because many representational forms beyond Lewis formulas are not explicitly mandated or are referenced only generically, whether and when they appear in lessons depends on teachers’ professional judgment within the curricular framework. Hence, in this study we focus on less clear but frequently occurring mixed forms between low-structured formulas, Lewis formulas, and valence-line formulas, as these mixed forms have the potential to confuse students, even though many textbooks and even teachers use a variety of mixed forms, for example, to illustrate the transition from one form of representation to another.^3^ Moreover, textbooks strongly influence teachers’ lesson planning and selection of representational forms.^26–28^

Research has shown that textbook usage of chemical representations is often inconsistent, both in terms of representational accuracy and in scaffolding representational competence.^29^ For example, an analysis of five widely used U.S. organic chemistry textbooks found that, although foundational representational skills such as interpreting and translating representations are introduced, there is often insufficient scaffolding in worked examples and practice problems to support students in acquiring these skills across different representational forms.^29^ Moreover, some commonly used representational forms—such as wedge–dash diagrams or condensed formulas—are introduced without clear explanation or rationale, and higher-level metarepresentational skills are rarely addressed.^29^ This patchy support can lead to confusion, especially when textbooks introduce structurally ambiguous or mixed representations without guidance. Similarly, chemistry textbooks rarely require students to translate between representations or interpret symbolic and particulate forms, further limiting opportunities to develop representational fluency.^30,31^ Some textbooks show inadequate links between macroscopic, submicroscopic and symbolic representations.^32,33^ These findings support the concern that students are not consistently supported in developing a coherent understanding of representation use, particularly when it comes to transitioning between representational forms.^34^ Accordingly, the abbreviations given should be understood as an aid to recognition and not as a definition of the notation.

Overall, it can be stated that the terminology used in textbooks deviates from IUPAC recommendations, varies between different textbooks, and is sometimes applied inconsistently even within a textbook (due to its dependence on the specific subject area). This clearly complicates the well-structured introduction of structural forms, the identification of errors when drawing formulas, and the support of students in learning how to purposefully read and draw structural formulas.

The same applies to three-dimensional representations at the submicroscopic level. Many students find it difficult to use their reasoning skills when dealing with abstract representations of scientific concepts whose understanding requires a high degree of spatial visualization^35,36^ (Figure 3).

**Figure 3:**
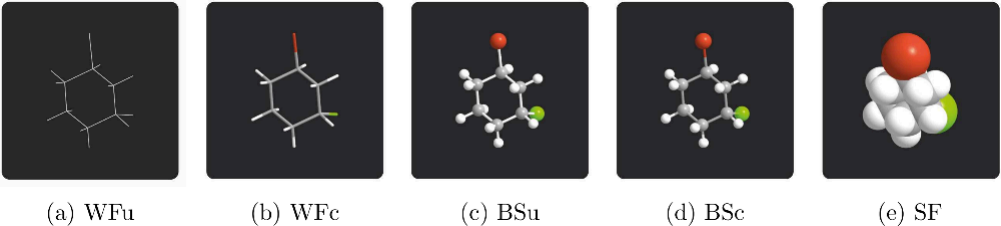
Three-dimensional representations of (1*R*,3*R*)-1-bromo-3-chlorocyclohexane: (a) wire-frame model with uncolored bonds (WFu), (b) wire-frame model with colored bonds (WFc), (c) ball-and-stick model with uncolored bonds (BSu), (d) ball-and-stick model with colored bonds (BSc), (e) space-filling model (SF).

While experts can translate fluently between multiple representations,^37–39^ as well as between macroscopic, symbolic and submicroscopic levels,^40^ novices must first build up the mental models required for this.^13,37,41^ This is a process that must be scaffolded from the outside^12,34,41–43^ (e.g., by using multiple representations^12,41,43,44^ and supplantation^45–47^ ).

## Conceptual Frameworks

This research is grounded in Johnstone’s conceptualization of representational knowledge domains^48–50^ and Kozma and Russell’s framework of representational competence,^12^ and is further extended by the three-factor model of representational competence proposed by Ward et al.^51^ (Figure 4).

**Figure 4:**
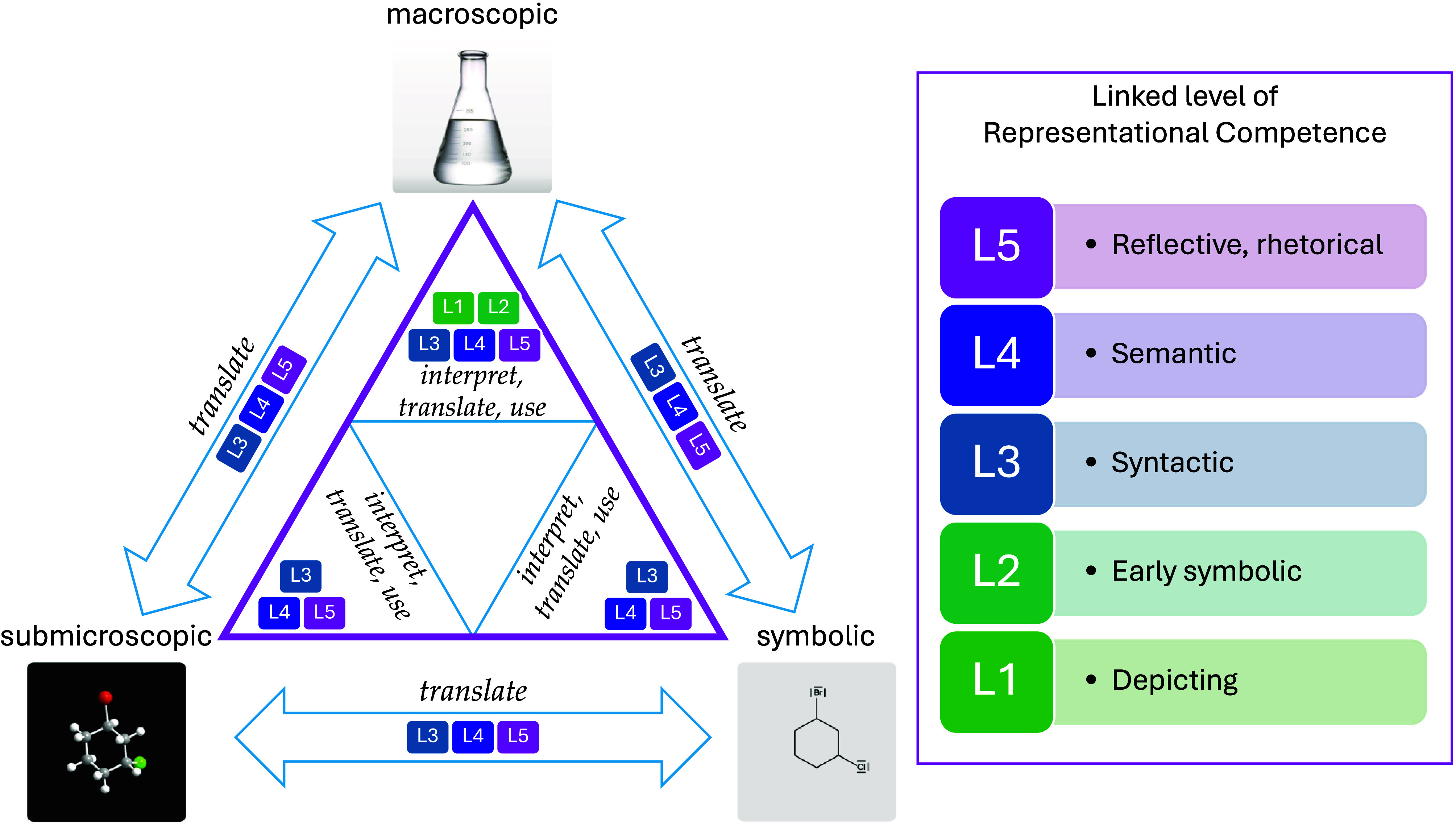
Author-created schematic illustrating the interplay of Johnstone’s triangle,^48,49^ linked levels of representational competence (RC),^12^ and Ward et al.’s three factors of RC skills.^51^ Erlenmeyer flask photo used under license from iStock; credit: iStock.com/studiocasper (Photo ID 177087121).

### Johnstone’s Triangle

Johnstone suggested that students must be able to understand chemical phenomena at several different levels to develop a coherent understanding of chemistry.^48–50^ This concerns the macroscopic level, on which visible chemical and physical phenomena can be observed, the submicroscopic level, on which chemical particles are located that are not visible to the naked eye, and the symbolic level, on which symbols and signs are used to represent macroscopic and submicroscopic phenomena. The relationships between these three levels are illustrated by Johnstone’s triangle (see triangle in Figure 4). Accordingly, chemistry teachers should avoid focusing too much on just one level and instead help students to first establish connections between two levels (the sides of the triangle) and then coordinate all three levels in their minds. Johnstone emphasized the importance of helping students recognize the differences between the levels by understanding each level individually and learning to translate between them in a targeted manner.^50^ The symbolic level is closely related to formal chemical terminology, which enables communication about chemical concepts at the macroscopic and submicroscopic levels.^52,53^ In organic chemistry, for example, symbolic reaction mechanisms, supplemented by the electron-pushing formalism, are used to explain interactions between particles in the submicroscopic realm, which in turn cause visible changes in the macroscopic realm. Teachers must therefore ensure that students not only correctly interpret letters, lines, and arrows in reaction equations, but also develop accurate mental models of the underlying macroscopic and submicroscopic processes.^53^ The development of expertise in the three areas of Johnstone’s triangle and the ability to translate between them are closely linked to the development of representational competence.

### Representational Competence

Kozma and Russell define *Representational Competence* (RC) as the “set of skills and practices that allow a person to reflectively use a variety of representations or visualizations, singly and together, to think about, communicate, and act on chemical phenomena in terms of underlying, aperceptual physical entities and processes”,^12^ p. 131. Accordingly, the RC framework^12^ outlines the competencies required to purposefully select from a variety of external representations or visualizations and to use them in a reflective way for scientific inquiry, presentation, communication, and scientific judgment.^6,12^ RC involves actively connecting observable chemical phenomena with the underlying, imperceptible physical entities and processes. Choosing or designing a representation to foster chemical understanding imbues that representation with meaning and reflects an individual’s level of RC. The expression of RC becomes particularly evident when comparing novices and experts. Whereas novices tend to rely on fewer representational forms and interpret them primarily based on surface features^6,54–58^ or rely on heuristics,^59^ and the application of symbolic rules,^60^ experts flexibly employ a wide range of representational forms to emphasize key ideas, support arguments, predict outcomes, solve problems, and explain complex phenomena.^5,6,12,56,61–65^

Kozma and Russell describe a set of seven skills that constitute RC,^12^ which can be grouped into three latent factors: the ability to *interpret*, *translate*, and *use* representations^51^ (italic text in Figure 4). They also propose five progressive levels of RC,^12^ describing a development from surface-based usage to reflective, rhetorical deployment (rectangles in Figure 4):^12,54,56^

- **Level 1 – Depicting:** Generation of representations as isomorphic, iconic images based on physical properties.
- **Level 2 – Symbolic:** Generation of representations as isomorphic, iconic images based on physical properties, incorporating individual symbolic elements. Representational forms are mainly used at the surface level, without reference to syntax or semantics.
- **Level 3 – Syntactic:** Generation of representations based on a formal symbol system with attention to syntactic rules.
- **Level 4 – Semantic:** Use of formal symbol systems grounded in syntactic and semantic rules and meanings to describe unobservable entities and processes with reference to physical phenomena. Establishment of links between two different representations and translation from one representation to another based on common meaning. Selecting the appropriate representational form for a specific purpose.
- **Level 5 – Rhetorical:** Deliberate use of representational forms to justify claims in a social or rhetorical context. This includes selecting or constructing the most appropriate representational form for a given situation and articulating why it is preferable over alternatives. It reflects an epistemological stance that representations provide structured access to otherwise unobservable knowledge.

It is important to note that these levels are not strictly discrete; transitions between them are fluid, and individuals may demonstrate different levels of competence across different domains and different representational forms. A student may demonstrate high proficiency with one representational form (such as Lewis formulas) while exhibiting low proficiency with another (such as Fischer projections).^12^

The RC framework is grounded in both the Cognitive Theory of Multimedia Learning (CTML)^12,66^ and situative theory.^12,67,68^ While CTML emphasizes learning as the result of processing and integrating visual and auditory information,^66,69^ situated learning theory views learning as a socially and communicatively embedded activity, in which scientific inquiry unfolds through collaborative processes, and is shaped by the social setting.^12,67,68^ Within the RC framework, collaborative scientific inquiry and representational forms influence one another. On the one hand, scientific progress shapes shared representational conventions; on the other, the norms of communication and representation actively shape how scientific inquiry is conducted.^12^ RC and conceptual understanding are interdependent at the individual level: foundational chemical knowledge is necessary to interpret representations, and representations, in turn, actively support the construction of new knowledge.^12,70,71^ Accordingly, Schönborn and Anderson highlight three interrelated factors that influence learning with representations:^70,71^ the student’s conceptual knowledge, overall reasoning ability, and the characteristics of the representational form.

Without basic subject-specific RC, students can neither successfully complete a chemistry degree^72,73^ nor pursue a profession related to chemistry.^11,12^ RC is fundamental to understanding core chemical concepts. Research shows that difficulties in chemistry often stem not only from conceptual misunderstandings but also from an inability to connect representations to the underlying conceptual information,^74–76^ and to move fluently between representational forms.^57,70,77^ The ability to interpret and apply external representations depends on cognitive factors such as prior knowledge and reasoning ability, as well as on explicit instruction and practice with representational forms.^70,77^

Although RC is widely recognized as an anchoring concept in chemical education,^9,12,13,70^ many instructors do not explicitly teach or assess these skills.^10,11,34,78^ Its absence, however, constitutes a significant barrier to learning. RC is what enables experts to move beyond surface-level interpretation and strategically select and use multiple representations to explain, and predict chemical phenomena.^12^ Moreover, combining different representations can have a positive influence on student learning.^79,80^ Instruction that targets representational reasoning has been shown to significantly improve students’ learning outcomes and conceptual understanding.^81^ RC is therefore not a peripheral skill, but a central element of chemical thinking, and a prerequisite for meaningful engagement in the discipline.

Multimedia tools can be used as learning aids to create and manipulate visualizations of molecular structures.^82–148^ The effectiveness of these three-dimensional visualizations is well-researched, and numerous studies have shown that the use of such visualization models can lead to significant improvements in understanding the underlying chemical processes and concepts.^34,85–88^ While these studies differ in their specific designs, target groups, and the software and visualization methods used, they all conclude that three-dimensional visualizations of molecular structures can be highly beneficial for understanding submicroscopic phenomena and processes—provided that the models are accompanied by appropriate instructional support.

Recent research shows the potential of augmented reality (AR) to advance representational competence and learning effectiveness, extending beyond the limitations of static 3D-models.^83^ AR allows students to interactively manipulate molecular structures and reaction mechanisms in real-world contexts, creating connections across the macroscopic, submicroscopic, and symbolic levels of representation.^89^ Research indicates that such environments can facilitate representational reasoning and helps students develop a clearer, more precise understanding of complex spatial concepts, such as chirality, while maintaining manageable levels of cognitive load^90^ (for an introduction to Cognitive Load Theory see^91,92^ ). Prior studies demonstrate that AR-based scaffolds facilitate students’ transitions between symbolic representations and spatial molecular geometry, thereby strengthening conceptual integration.^93^ Moreover, research on lesson design underscores that AR is most effective when carefully embedded in instruction, with explicit objectives and a clear alignment between AR activities and learning outcomes.^93^ Altogether, these findings suggest that AR not only increases motivation but also substantially improves the acquisition and application of representational competence, provided it is integrated into coherent and pedagogically structured learning contexts.

## Aims and Research Questions

This study is conceptually embedded in the OrChemSTAR project, which develops and evaluates learning environments for reading and drawing chemical structures. In this article, we report only the teacher-focused needs assessment; for project context, objectives, target populations, and planned evaluations, see SI, Section 5. With this study, we contribute to the clarification of how chemistry teachers assess the typical errors that students make when drawing structural formulas as well as how chemistry teachers usually introduce representational forms in class based on which chemical compounds. Additionally, we investigate how students’ misconceptions that hinder the drawing of formulas can be diagnosed, and how teachers counteract these difficulties in class. The main focus of this study is on teachers who teach in upper secondary education at Swiss Matura schools. In order to be qualified to teach at a Swiss Matura school, both a relevant and complete subject-specific university degree and a postgraduate teaching diploma are required. Many of these teachers have attained doctoral degrees in their respective subject areas and/or have accumulated professional experience in industry and business. Therefore, depending on their specialization, these teachers are likely to be influenced not only by curricula and textbooks, but also by their academic and professional backgrounds—factors that significantly shape their use of external representations in the classroom (for the conceptual framework, see SI, Section 4). Therefore, the personal perspectives of these teachers constitute the focal point of this research.

Accordingly, we investigate the following research questions in this study:

**RQ1.1:** At what point in schooling do teachers expect students to first be able to *interpret* different representational forms of chemical structures, and when do teachers expect students to first be able to *produce* these forms independently? *(Timing of introduction of representational forms)*

**RQ1.2:** Which chemical compounds do teachers typically use to *introduce* these representational forms? *(Compounds used to introduce representational forms)*

**RQ2.1:** What typical errors do teachers observe in their students’ work when students *produce* these representational forms? *(Common student errors when producing representational forms)*

**RQ2.2:** Which chemical compounds do teachers use to *diagnose* these errors? *(Compounds used to diagnose common errors)*

**RQ3:** What support strategies do teachers report using to address typical student errors? *(Strategies to address common errors)*

## Methods

To investigate when, in the opinion of practicing teachers, students should be able to read and write various representational forms (RQ1.1), which chemical compounds are used to introduce these representational forms (RQ1.2), what typical difficulties teachers observe in their students (RQ2.1), and which compounds are particularly suited to diagnose such difficulties (RQ2.2), an online survey was conducted. To address the third research question—how teachers support their students in overcoming these typical difficulties (RQ3)—a series of online interviews was conducted. Respondents answered with reference to their own teaching practice and professional judgment in their current setting, recognizing curricula and internal school syllabi as boundary conditions.

### Sample

Of the 197 Swiss, German, Austrian and Danish chemistry teachers invited, 116 took part in the survey (45 female, 64 male, 1 diverse, 3 no response; aged 44(10.7) years; 99 Swiss, 13 German, 1 Danish, 6 no response). Table 1 lists the qualifications of participants.

**Table 1:** Qualification of Participants^a^

Qualification	Count
Degree in chemistry (e.g., Diploma)	59
Degree in nonchemistry natural science subject	25
Doctorate in chemistry	30
Doctorate in chemistry education	4
Doctorate in nonchemistry natural science subject	12
Doctorate in nonchemistry natural science subject education	2
Doctorate in other subject	0
Teaching diploma in chemistry for higher secondary education	81
Teaching diploma in chemistry for lower secondary education	3
Teaching diploma in other natural science subject	18
German state exam for higher education	19
German state exam on another educational level	0

a Multiple answers allowed.

At the end of the survey, participants were asked whether they would be available for a follow-up interview. Of the 116 participants, 18 agreed, and ultimately 11 interviews were conducted during the data-collection period. Across all sociodemographic data and questionnaire responses, no statistically significant differences were found between those who were actually interviewed and those who were not (including both participants who declined and those who agreed but could not be scheduled) (Holm-adjusted *p* ≥ 0.05). Effect sizes were generally small, suggesting no systematic bias in the interview subsample.

This study adhered to ethical guidelines in accordance with European, national, and cantonal regulations as well as institutional policy. The sample of Swiss chemistry teachers was compiled by collecting publicly available e-mail addresses from Swiss matura school websites. To supplement this sample, additional teachers from Germany, Austria, and Denmark—already known to the research team—were invited to participate. All participants took part voluntarily. Prior to the online survey, participants were informed about the purpose of the study, the nature and scope of data collection, the measures taken to ensure anonymity, and their right to withdraw consent at any time before full anonymization. Informed consent was obtained electronically. At the conclusion of the survey, participants were invited to take part in follow-up interviews. Interviewees were again informed about the study’s objectives and procedures, and provided informed consent to audio and video recording and the use of transcription services. As the collection of personal data was minimized and the research posed negligible risk to participants, no approval from an ethics review board was required under applicable legal and institutional frameworks.

### Instruments

#### Questionnaire

RQ1.1–2.2 were addressed with a questionnaire specifically developed for this study. After reading the privacy notice and providing consent to data processing, participants were welcomed again and reminded that their responses should refer to their own teaching practice, with particular reference to the use of structural formulas in their chemistry lessons and to their experiences with student learning (for the introductory text, see SI, Section 6, Table S4). Participants were first asked to provide demographic information, including gender, year of birth, education, place of work, school levels taught, professional experience, and classes taught in the current school year. Subsequently, participants responded to eight items based on the prompt: “For each of the representational forms shown below, please indicate when your students should be able to read them for the first time and when they will need to be able to create them themselves for the first time.” The questionnaire displayed the exact same representations as those shown in Figure 2—such as molecular formulas, condensed formulas, skeletal formulas, and wedge–dash formulas—with a particular focus on less common, but regularly found in textbooks, mixed forms of standard representational forms, such as skeletal formulas with lone pairs in dot notation and explicit hydrogen atoms. Participants were asked to rate, based on their professional judgment, a) in what grade students should first be able to read these representational forms, and b) in which grade they should be able to create these representational forms themselves for the first time. Responses were collected using two separate eight-point rating scales from “Grade 7–” to “Grade 13–” plus “never”.

For each representational form that teachers reported using in their own courses, it was also asked (via conditionally displayed items) a) which chemical compounds teachers usually use to introduce these representational forms in class, b) which typical errors students often make when creating these representational forms and c) which simple chemical compounds can be used to easily recognize these typical errors. These follow-up items were shown only for representational forms the respondent had indicated using. Responses were collected using open-ended questions.

In addition, participants were asked to indicate from which school level students should be able to represent the structures of 18 selected chemical compounds (listed in Table 2). All compounds were taken from prior research,^57^ except for H_2_O, C_3_H_8_, and C_2_H_2_. For each compound, participants indicated the school level at which students should be able to represent it (a) without an explicit spatial arrangement (e.g., Lewis formula or line formula), and (b) with an explicit spatial information (e.g., wedge–dash notation or Natta projection).

**Table 2:** Time (in Measures of School Grade) of Introduction of Selected Exemplary Chemical Compounds

Compound	Without spatial information	With spatial information
H_2_O	9.2	9.6
C_3_H_8_	9.7	10.1
C_2_H_2_	9.7	10.1
CH_3_OH	9.7	10.2
CH_2_O	9.7	10.2
CH_4_S	9.7	10.2
HCN	9.8	10.0
C_2_H_6_O	9.8	10.3
CH_4_O	9.8	10.3
CH_3_COOH	9.9	10.3
C_3_H_7_NO	9.9	10.5
C_2_H_4_O_2_	9.9	10.4
NO	10.3	10.6
NH_2_^–^	10.4	10.6
C_2_H_5_O^–^	10.6	10.7
C_2_H_3_O_2_^–^	10.6	10.7
CH_6_N^+^	10.7	10.8
CH_5_O^+^	10.7	11.0

Finally, participants were again asked, analogous to the first part of the questionnaire, to assess the school level at which students should be able to a) read and b) create various types of projection formulas (e.g., Haworth, Fischer, Newman, etc.)—exactly those five representational forms shown in Figure 1. Responses were again collected using two separate eight-point rating scales from “Grade 7–” to “Grade 13—” plus “never”.

#### Interview Guide

The follow-up interviews were conducted using a semistructured interview guide to ensure comparability between interviewers and participants. The interviews were based on six guiding questions:

1. What typical or frequently occurring difficulties and errors do you generally find students make when using structural representational forms? This refers to both reading and creating structural formulas.
2. What other, possibly rare, student errors, difficulties or challenging concepts related to structural representational forms should we consider?
3. What other student perceptions or misconceptions hinder the learning of the presentation methods?
4. Please describe how you deal with these errors, difficulties and misconceptions and how you proceed in providing individual support to students.
5. Which chemical compounds are particularly suitable for diagnosing students’ difficulties and detecting errors?
6. Can you give further examples of where it would be or is helpful to support students in learning how to write structural formulas? If necessary, please also explain how you do this.

For each of these guiding questions, interviewers were allowed to ask one or more of the following prompting questions to encourage participants to elaborate further:

- Can you draw this example and show it to me?
- Is there anything else to note?
- Are there any other examples (or situations)?
- How would that be helpful?

### Study Design

The online survey was conducted in February 2024 using LimeSurvey.^94^ All chemical practitioners in German-speaking Switzerland with publicly accessible e-mail addresses were invited to take part in the survey. In addition, the survey was advertised via national professional associations. The test group was supplemented by selected German, Austrian, and German-speaking Danish chemistry teachers.

The online survey was supplemented by a guided online interview (30–45 min), which was conducted as a video conference and during which images and sound were recorded.

### Safety Statement

No unexpected or unusually high safety hazards were encountered in this study. The work involved an online survey and video-conference interviews with teachers and did not include laboratory activities, chemicals, or hazardous equipment.

### Data Analysis

The survey data were analyzed using the statistical software R.^95^ To determine the grade levels at which students, according to the surveyed teachers, should be able to read and draw the presented structural and projection-based representational forms (RQ1.1), heat maps were created using the ggplot2 ^96^ and cowplot^97^ packages. In addition, for selected compounds, the average grade (as reported by participants) at which students should be introduced to the compounds—both without and with spatial information—was calculated based on the respective rating scale. “Never” responses were excluded from mean grade calculations (treated as missing for the mean) but are reported descriptively.

To evaluate the frequency of terms used in open-ended responses regarding frequently used compounds for introducing representational forms (RQ1.2) and diagnosing common errors (RQ2.2), a semiautomatic qualitative content analysis was conducted. First, all responses across the relevant questions were tokenized into single words, and word frequencies were calculated. In the second step, beginning with the most frequent terms, all words referring to the same compound (including synonyms, retained names, molecular formulas, typos, etc.) were grouped until a total of 101 unique compounds was identified. These compounds were then categorized into broader chemical groups (e.g., alkanes, alkynes, haloalkanes, etc.). Frequencies were then determined both by compound and by category for each representational form and for both research questions independently.

To identify the typical errors students make, as perceived by teachers (RQ2.1), a standard qualitative content analysis (QCA) approach with inductive category development was applied. Starting from a set of known potential errors, all responses to the relevant open-ended question were analyzed, categorized, and expanded with additional categories as needed. All answers were then recoded using the finalized category rubric by two independent raters, with full agreement achieved. Frequencies were calculated for all error categories by representational form.

To assess potential selection effects, we compared interviewees with noninterviewed participants (including those who declined or could not be scheduled) across all available sociodemographic data and questionnaire responses using chi-square tests for categorical variables and Wilcoxon rank-sum tests for ordinal or numeric variables, applying Holm’s correction for multiple testing.

### Interview Analysis

The evaluation of the interviews (RQ3), which aimed to understand the support strategies teachers use to help students overcome difficulties, was carried out in our study and concurrently served a methodological purpose: a coauthor’s master’s thesis benchmarked the quality of artificial intelligence (AI) tools for transcription and coding of interviews that contain substantial technical language.^98^ We therefore used the same interview corpus to assess AI-supported steps at different stages of the analysis workflow. To ensure comparability, the interviews were first transcribed both manually and using the AI-based tool noScribe.^99^ These two transcripts were then consolidated into a unified version that served as the basis for all subsequent analyses. The consolidated transcript was analyzed in parallel using the following methods:

- Human-based qualitative content analysis.^100^
- AI-based qualitative content analysis using ChatGPT-4o.^101^
- AI-based qualitative content analysis using MAXQDA Analytics Pro (version 24.6.0).^102^
- Human-based narrative analysis.^103^
- AI-based narrative analysis using MAXQDA Analytics Pro (version 24.6.0).^102^
- AI-based narrative analysis using ChatGPT-4o.^101^

Our dual-purpose design is pragmatic: alongside addressing RQ3, the same interview corpus allows us to benchmark AI support for transcription and coding in a technically specialized domain. Strengths include direct comparability via dual transcription (manual and AI) before consolidation, methodological triangulation through parallel human and AI analyses (content and narrative), and reproducible, versioned workflows that leave an audit trail. Limitations include potential AI errors with domain-specific jargon, sensitivity to prompts and model/software versions, and the need to prevent any priming of human coders by AI outputs. To mitigate these risks, human coding was conducted independently and remained decisive; AI outputs served as a supplementary comparison rather than a replacement for human analysis.

Finally, a synthesis of the results from all analysis methods was carried out to summarize the key conclusions related to RQ3.

## Results

### Timing of Introduction of Representational Forms (RQ1.1)

To compare the grades in which teachers believe students should first be able to read or draw specific representational forms, we visualized the frequencies of responses as heat maps (Figures 5–8). The heat maps provide an overview of both the most frequent responses and their distribution across grades. Representational forms are arranged in ascending order by mean reported grade, so their position on the *x*-axis reflects the average sequence of introduction.

**Figure 5:**
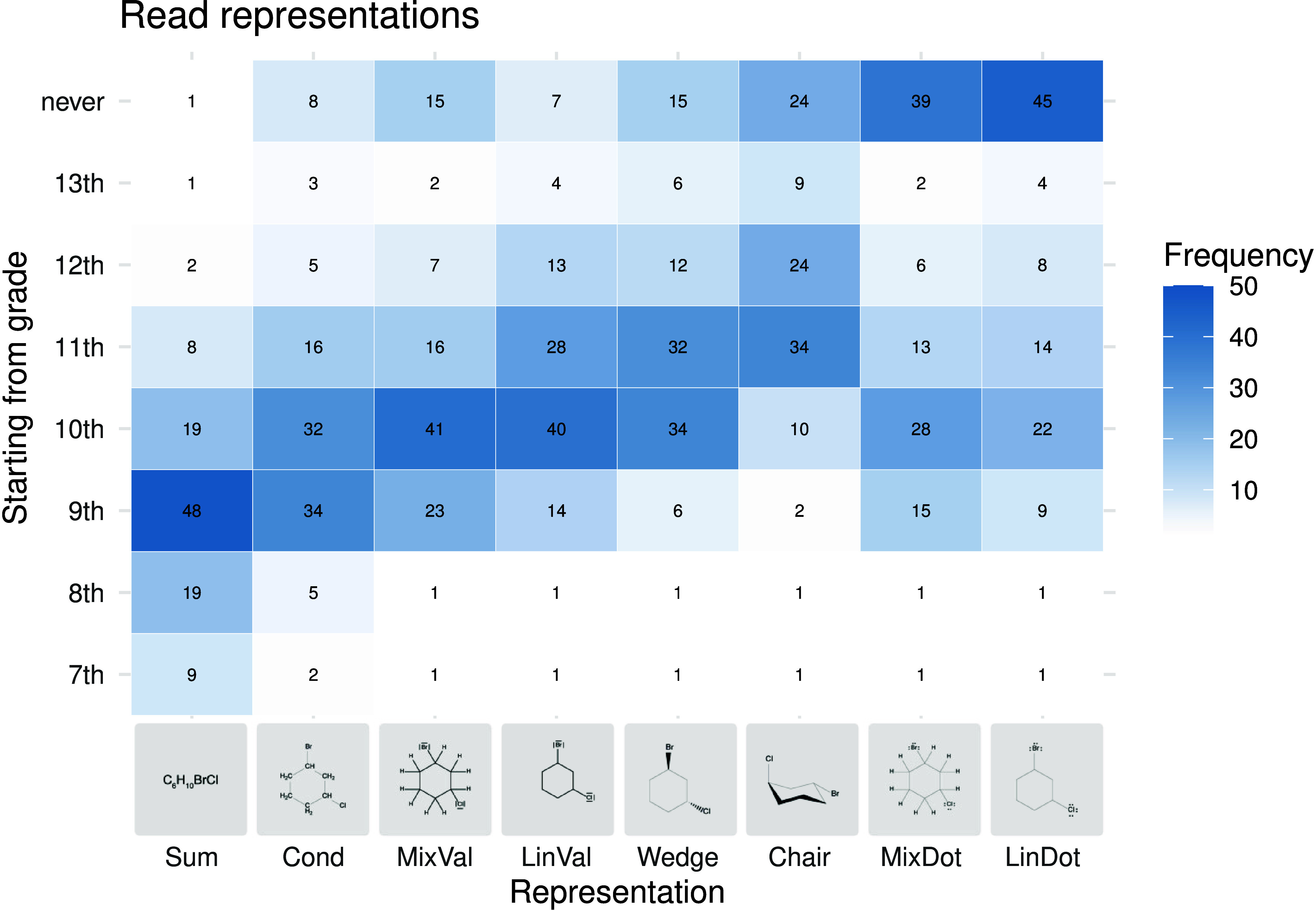
Heat map of the grades from which students should be able to read the respective representations of chemical formulas according to the surveyed teachers.

Teachers reported that, in the enacted curriculum, students are first expected to read and write molecular formulas, followed by condensed (semistructural) formulas. Skeletal formulas with lone pairs in line notation are expected to be mastered before the corresponding electron dot variants. Variants with explicit hydrogen atoms are expected to be mastered before the corresponding variants without explicit hydrogen atoms. For representations with additional information on the spatial arrangement, wedge–dash formulas are expected to be mastered before representations of cyclohexane conformation. The order of reading ability (Figure 5) and drawing ability (Figure 6) is identical but the students should usually be able to read the representational forms before they draw them.

**Figure 6:**
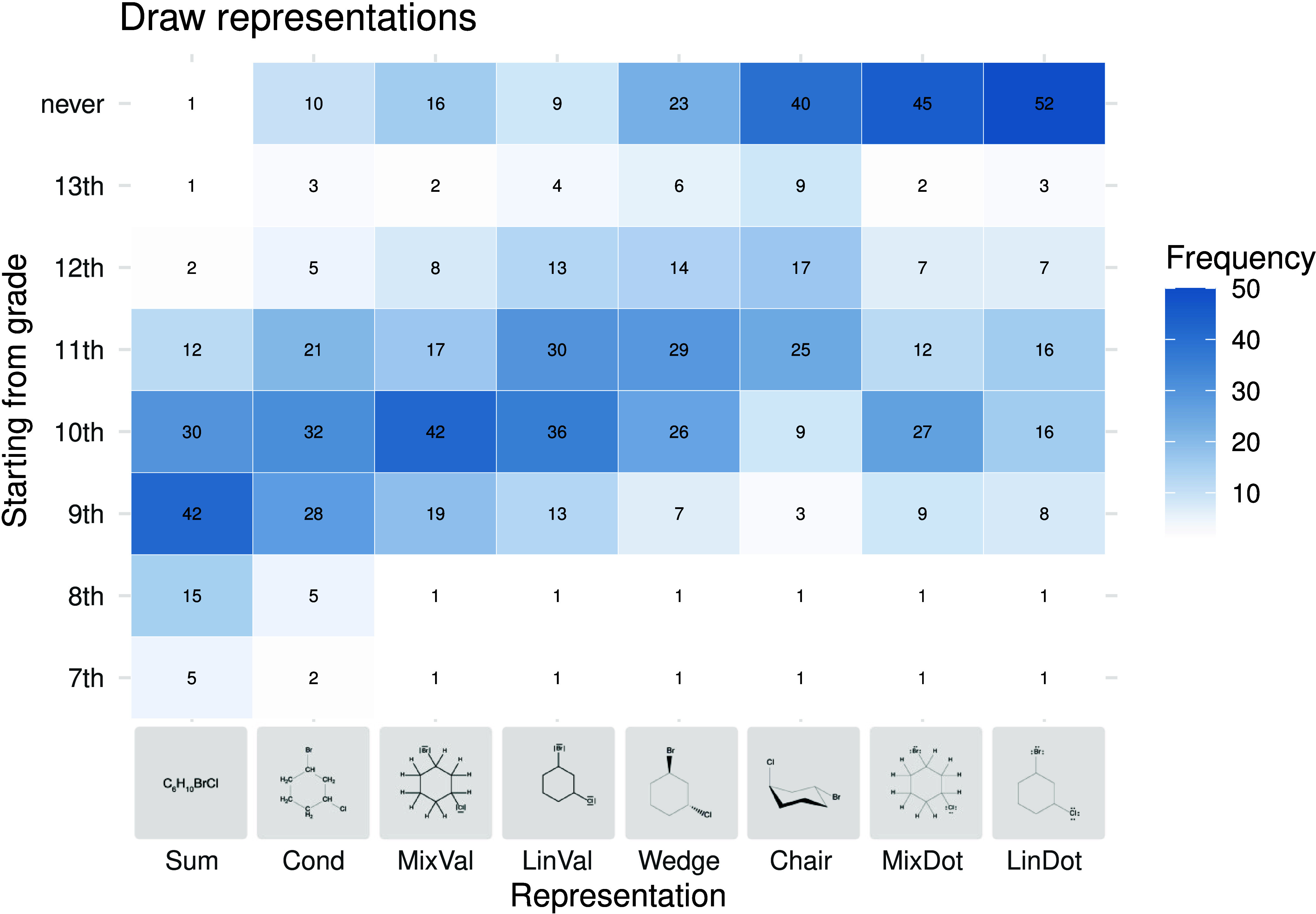
Heat map of the grades from which students should be able to write/draw the respective representations of chemical formulas according to the surveyed teachers.

The findings on the projections for the representation of the spatial arrangement of atoms in sugar molecules are consistent with the observations on the more general structural formulas. The spatial information on sugar molecules is also initially represented using Mills projections (Figure 7). Later, students should also be able to read (Figure 7) and draw (Figure 8) Haworth projections as well as cyclohexane conformations. In the case of the Fischer projection, there are two opposing positions among the teachers surveyed. While 21 and 27 teachers introduce this representational form in 11th and 12th grade, respectively, 33 teachers (i.e., 36% of valid responses) state that students should not be able to read the Fischer projections at all at school. Regarding writing this form, 50% do not introduce it. For the Newman projections, as many as 79% for reading and 82% for writing state that students do not need to have the relevant skills.

**Figure 7:**
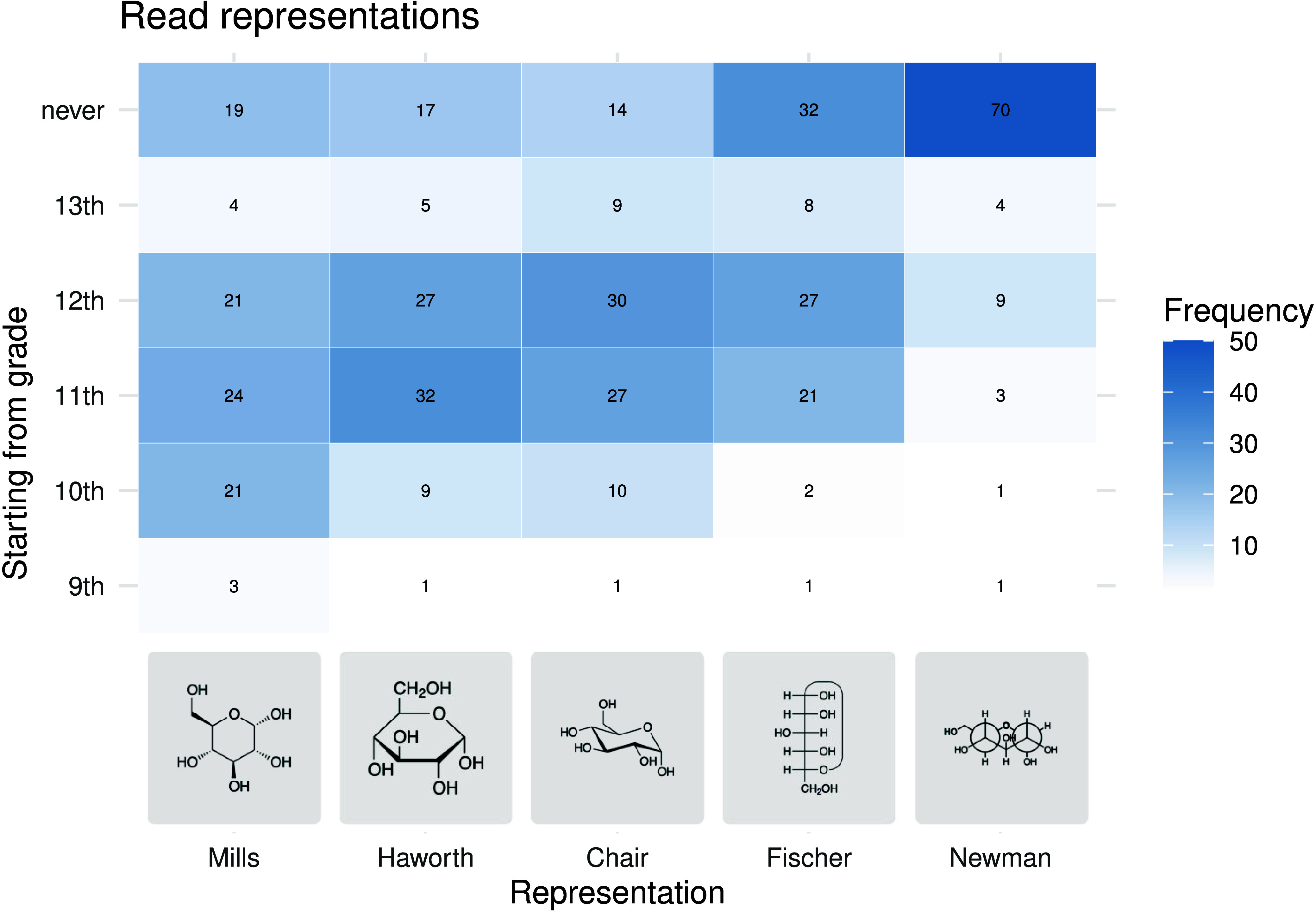
Heat map of the grades from which students should be able to read the respective projections according to the surveyed teachers. (No answers for grades 7 and 8.)

**Figure 8:**
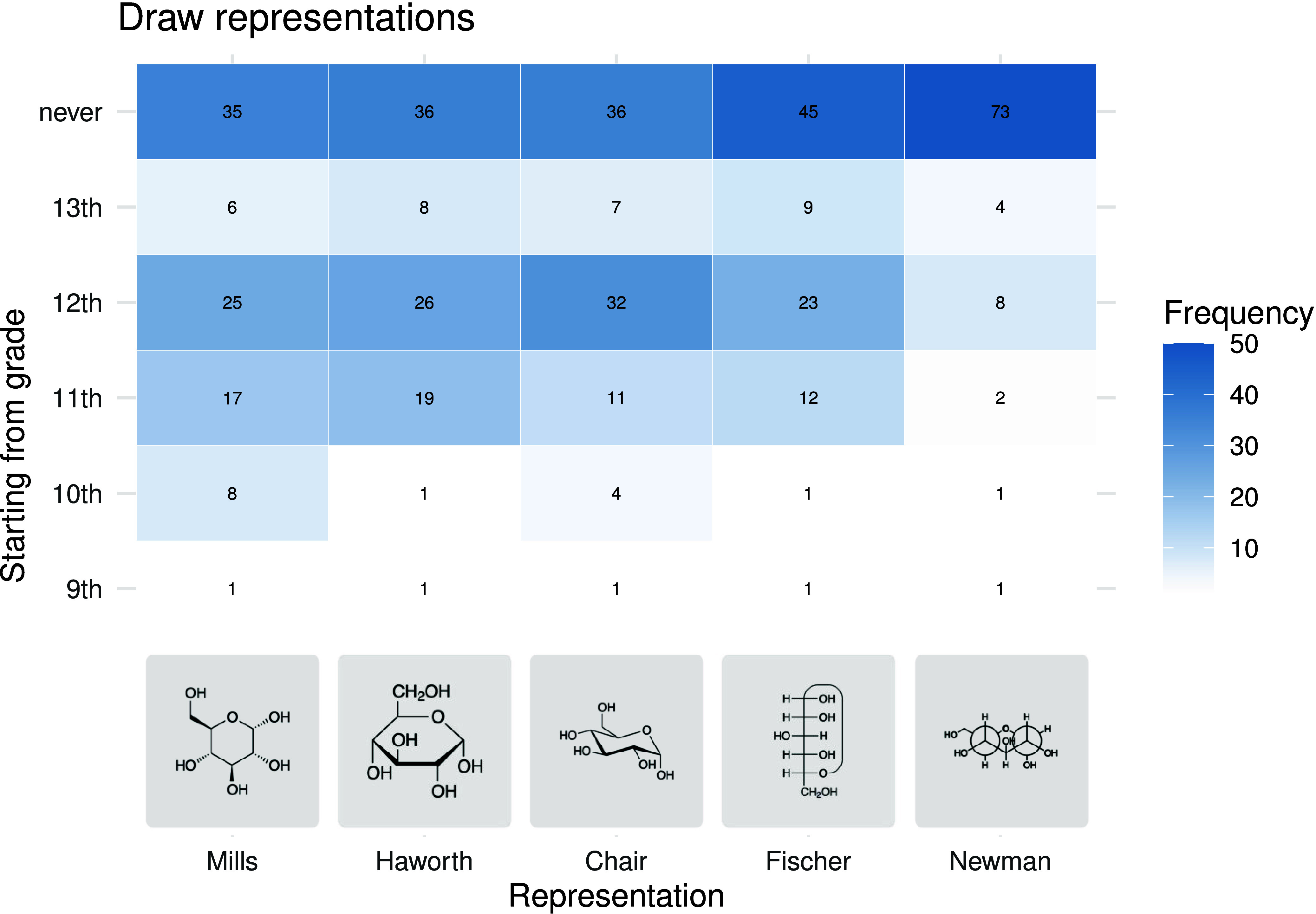
Heat map of the grades from which students should be able to draw the respective projections according to the surveyed teachers. (No answers for grades 7 and 8.)

In addition, participants were asked at which grade level they first introduce selected chemical compounds commonly used in school lessons. They were asked about representations with and without spatial information. Table 2 lists the average year groups in which the compounds mentioned were introduced. It is visible that the compounds are consistently first introduced without spatial information and later with spatial information. Furthermore, the time between the introduction of the compound without spatial information and the introduction of the compound with spatial information decreases as the grade of the students increases.

### Compounds Used to Introduce Representational Forms (RQ1.2)

The participants were asked which chemical compounds they used to introduce these notations for each of the representational forms that they used in class. They could also specify several compounds or substance classes in a free text field. Table 3 lists the most frequently mentioned compounds and indicates the number of mentions per representational form (for a full list of compounds, see SI, Section 7, Table S5). Overall, alkanes and alcohols were the most frequently reported substance classes for introducing different representational forms, with methane, ethane, and ethanol being particularly prominent. Inorganic compounds such as oxygen, hydrogen, and nitrogen were also used regularly, especially in the introduction of molecular formulas (Sum). Sugars, mainly glucose, were mentioned less frequently, followed by carboxylic acids, cycloalkanes, and haloalkanes. Other substance classes (e.g., aldehydes, alkenes, aromatics, ethers) were rarely mentioned.

**Table 3:** Main Categories of Frequently Used Chemical Compounds to Introduce Different Notations^a^

Compound	Sum	Line	MixVal	LinVal	Wedge	Conf	MixDot	LinDot	Total
Alkanes	28	26	8	11	1	11	16	13	114
Alcohols	15	13	3	11	6	11	3	5	67
Inorganic compounds	14	3	9	11	4	1	0	0	42
Sugars	12	1	0	0	0	1	2	21	37
Carboxylic acids	3	7	4	6	1	3	1	1	26
Cycloalkanes	1	7	2	0	1	5	2	6	24
Haloalkanes	2	1	4	2	1	7	2	3	22
Aldehydes	2	1	0	3	0	1	0	1	8
Alkenes	0	1	0	0	0	4	3	4	12
Aromatic compounds	0	2	0	0	0	1	1	1	5
Ethers	1	1	1	1	0	1	0	0	5
Halocycloalkanes	1	1	0	0	0	1	0	0	3
Alkynes	0	1	0	0	0	3	0	1	5
Alkaloids	2	0	0	0	1	0	0	0	3
Ketones	0	0	1	0	0	1	0	0	2
Organic compounds	0	1	0	0	0	0	0	1	2
Hydrocarbons	0	0	0	0	0	0	0	0	0
Carbohydrates	0	0	0	0	0	0	0	0	0
Elements	0	0	0	0	0	0	0	0	0

a See SI, Table S5 for full list of compounds.

### Common Student Errors When Producing Representational Forms (RQ2.1)

The questionnaire results reveal, based on teachers’ perceptions, several typical errors made by students when drawing structural formulas. Table 4 categorizes these errors and quantifies them across different representational forms. The most prevalent error categories, with a cumulative frequency of at least 25 mentions across all representational forms, include: incorrect bond (InBo), missing bond (MiBo), excess atom (ExAt), missing atom (MiAt), octet rule violated (Oct), incorrect electron (InEl), missing electron (MiEl), incorrect spatial orientation (InSp), incorrect indices (InIn), difficulties with syntax and transfer (Syntax).

**Table 4:** Typical Errors Made by Students When Drawing Different Representational Forms

Code	Category	Sum	Line	MixVal	LinVal	Wedge	Conf	MixDot	LinDot	Total
InBo	Incorrect Bond	2	8	6	3	1	2	4	2	28
ExBo	Excess Bond	1	10	8	0	1	2	3	2	27
MiBo	Missing Bond	1	10	7	0	1	5	3	7	34
InAt	Incorrect Atom	4	1	0	1	0	4	0	0	10
ExAt	Excess Atom	7	10	6	2	4	8	3	7	47
MiAt	Missing Atom	10	13	7	5	4	12	5	7	63
OrAt	Order of Atoms	14	1	2	0	0	0	0	0	17
InCh	Incorrect Charge	1	1	1	0	0	0	0	0	3
ExCh	Excess Charge	1	1	1	0	0	0	0	0	3
MiCh	Missing Charge	1	1	1	0	0	0	0	0	3
Oct	Octet rule violated	8	13	9	2	2	3	2	2	41
Hund	Hund’s rule ignored	0	0	0	1	0	0	0	0	1
InEl	Incorrect Electron	1	4	6	2	1	1	11	4	30
ExEl	Excess Electron	0	5	6	2	2	1	9	5	30
MiEl	Missing Electron	1	10	15	5	3	3	13	13	63
InSp	Incorrect Spatial Orientation	1	5	6	0	22	17	5	4	60
InIn	Incorrect Indices	25	2	3	1	0	0	0	0	31
MiBr	Missing Brackets	4	1	1	0	0	0	0	0	6
AtSym	Incorrect Atom Symbols	4	0	1	0	0	0	0	0	5
Group	Difficulties with Functional Groups	0	2	1	0	0	1	0	5	9
Syntax	Difficulties with Syntax and Transfer	6	2	5	4	4	9	2	13	45
Element	Difficulties with Elements	1	0	0	1	0	0	0	0	2
Drawing	Inappropriate Drawing	0	1	0	2	8	6	0	6	23

Because error categories are not equally applicable across representational forms, we summarize salient patterns within each form (and, separately, for the overall total) and do not compare frequencies between forms. Within molecular formulas (Sum), the most frequent issues were incorrect indices (InIn) and atom-order problems (OrAt), alongside missing/excess atoms and some octet-rule violations. Within valence-line formulas (Line), missing atoms (MiAt) and octet-rule violations (Oct) were most common, with many reports of missing/excess bonds (MiBo/ExBo) and missing electrons (MiEl). Within representations of cyclohexane conformations (Conf), incorrect spatial orientation (InSp) dominated, with additional mentions of missing/excess atoms and notation/syntax difficulties (Syntax). For skeletal formulas with explicit hydrogens, the emphasis shifted to electron accounting: in the line-notation variant (MixVal), missing electrons (MiEl) were most frequent, followed by octet-rule violations and bond-count errors; in the dot-notation variant (MixDot), electron placement/omission (InEl/MiEl/ExEl) predominated. For skeletal formulas with implicit hydrogens and lone pairs in line notation (LinVal), reports were more diffuse, with missing atoms/electrons and general syntax issues appearing most often. Within wedge–dash formulas (Wedge), incorrect spatial orientation (InSp) was by far the most prominent category, with drawing-execution issues (Drawing) also noted. For skeletal formulas with implicit hydrogens and lone pairs in dot notation (LinDot), missing electrons (MiEl) and notation/syntax difficulties (Syntax) were most frequent, with additional reports of bond-count errors (MiBo), atom omissions/excess (MiAt/ExAt), and some spatial-orientation and drawing issues.

Across all representational forms combined, the most commonly reported categories were missing atoms (MiAt, 63) and missing electrons (MiEl, 63), followed by incorrect spatial orientation (InSp, 60), excess atoms (ExAt, 47), notation/syntax difficulties (Syntax, 45), and octet-rule violations (Oct, 41). Bond-count errors were also frequent (MiBo, 34; ExBo, 27), as were electron placement/excess issues (InEl, 30; ExEl, 30). Incorrect indices (InIn, 31) were salient within molecular formulas but, by design, less applicable elsewhere. Less frequent overall were drawing-execution issues (Drawing, 23) and the remaining categories.

### Compounds Used to Diagnose Common Errors (RQ2.2)

The participants were asked which chemical compounds they frequently use to diagnose common errors for the given representational forms. Responses were entered in free-text fields, allowing participants to name compounds or substance classes. Table 5 lists the most frequently mentioned chemical compounds and indicates the number of mentions per representational form (for a full list of compounds, see SI, Section 7, Table S6 for a full list of compounds). Across representational forms, alkanes and alcohols were the most frequently reported for diagnosing common errors, with methane, ethanol, and related simple molecules appearing repeatedly. Carboxylic acids, especially acetic acid, were also mentioned relatively often, followed by sugars and some simple alkenes. In contrast, inorganic compounds such as hydrogen, oxygen, and nitrogen, as well as halogens, were only rarely reported. Overall, teachers tend to rely on smaller and structurally simpler molecules to elicit and diagnose typical errors, while more complex organic compounds (e.g., amines, aromatic systems, alkaloids) were almost never used.

**Table 5:** Main Categories of Frequently Used Chemical Compounds to Diagnose Common Errors by Notation^a^

Compound	Sum	Line	MixVal	LinVal	Wedge	Conf	MixDot	LinDot	Total
Alkanes	9	7	2	2	0	2	6	6	34
Alcohols	7	7	3	6	3	4	2	2	34
Carboxylic acids	5	5	2	5	1	5	3	1	27
Sugars	0	1	0	0	0	0	3	14	18
Alkenes	2	4	2	3	0	1	3	1	16
Inorganic compounds	6	0	0	2	0	0	0	0	8
Cycloalkanes	1	2	2	1	0	2	0	1	9
Haloalkanes	0	0	0	2	1	3	0	1	7
Aldehydes	2	0	0	2	0	1	0	0	5
Alkynes	1	1	1	0	0	0	1	1	5
Ethers	1	0	1	1	0	2	0	0	5
Aromatic compounds	0	2	0	0	1	0	0	0	3
Ketones	0	0	0	1	0	2	0	0	3
Halocycloalkanes	2	0	0	0	0	0	1	0	3
Alkaloids	2	0	0	0	0	0	0	0	2

a See SI, Table S6 for full list of compounds.

### Strategies to Address Common Errors (RQ3)

The findings from the teacher interviews revealed several recurring themes regarding common student difficulties and strategies that teachers use to address them.

#### Challenges Related to the Octet Rule and Valence Concepts

Many teachers reported that students often struggle with understanding the octet rule. According to interviewees, exercises are particularly beneficial in this context, especially those involving compounds with elements that can exhibit different valences (e.g., phosphorus or sulfur). Teachers emphasized that targeted exercises are helpful, especially when students are required to identify and correct intentional errors in chemical representations. For example, exercises might include incorrect Lewis formulas where the octet rule is violated, prompting students to justify the correction based on underlying rules. According to the teachers, such strategies are manageable for students even at introductory levels.

#### Importance of Repetition and Practice

Teachers consistently stressed the value of repeated practice. Repetition was said to reduce mechanical errors and reinforce conceptual understanding, especially in distinguishing between different representational forms. It would also help students improve their ability to translate between representational forms and to develop spatial reasoning. Therefore, interviewees recommended providing a large number of exercises that are organized by both their degree of difficulty and their conceptual focus. For example, differentiating representations of salts from representations of covalent molecules, or explicitly representing lone pairs, were mentioned as focal points.

#### Representational Complexity and Scaffolding

The valence-line formula was described by teachers as a central and important representational form. It is widely used and described by the interviewees as the basis for understanding other representational forms. However, skeletal formulas are considered the most challenging representational form for students to read and write. Comprehending the three-dimensionality of the molecules was said to be one of the biggest challenges for students. Some interviewees proposed the use of technology, such as Augmented Reality (AR), to support the three-dimensional representations of molecular structures. As an example, teachers described AR applications that could automatically display lone pairs, which are often forgotten or misunderstood by students. These suggestions were presented as aspirations or ideas for future support tools rather than established classroom practices.

#### Individual Learning Paths

Some teachers emphasized the importance of introducing representational forms in a sequence that reflects their increasing abstraction and complexity. Specifically, they described transitions from highly detailed 3D models—such as space-filling or ball-and-stick—to more schematic forms like skeletal formulas. According to teachers, this progression helps students gradually build representational fluency without being overwhelmed.

#### Compounds Used to Diagnose Difficulties

In the opinion of the interviewed teachers, simple molecules such as ethanol, hydrogen chloride, water, ammonia, or hydrogen sulfide would be considered suitable for the identification of errors and underlying misconceptions. Difficulties with the octet rule and the Lewis formula could, in their view, be recognized particularly well using sulfur dioxide and sulfur trioxide. The exploration of E/Z or cis/trans isomerism with double bonds could be conducted through the use of ethene with two distinct substituents. An understanding of three-dimensionality and spatial behavior, they suggested, could be effectively supported by using the dichloromethane molecule, as it adopts a tetrahedral geometry.

## Discussion

This study explored chemistry teachers’ perceptions of the difficulties students encounter when reading and drawing chemical structures. The findings must be understood within the Swiss curricular context. Swiss curricula focus on competencies and levels of abstraction rather than prescribing specific compounds or representational forms.^104–127^ Teachers thus exercise professional judgment in selecting substances and representational forms.^25^ This explains the observed emphasis on simpler molecules and a gradual progression to more complex forms. Our results therefore reflect the enacted curriculum, where curricular guidelines and teachers’ expectations intersect.^26–28^ We do not interpret differences between teacher judgments and curricular documents as noncompliance. Instead, they indicate how teachers respond when the curriculum lacks guidance on specific representational forms.

### Timing of Introduction of Representational Forms (RQ1.1)

Teachers expect students to interpret common representational forms, such as Lewis or valence-line formulas, in ninth or 10th grade. More abstract forms, such as skeletal, wedge–dash, or projection-based forms, are introduced later. Several teachers mentioned that wedge-and-dash bonds confuse students, often misreading them or ignoring them altogether. This reflects a well-documented challenge in chemistry education: novice students typically have limited spatial ability and struggle with visualizing and interpreting three-dimensional representations.^35,36^

Drawing representations consistently lags behind reading them by about one grade level. This finding supports previous work showing that representational competence develops progressively and depends on both exposure and instructional support.^12^ The gradual timeline aligns with Johnstone’s representational triangle model, which emphasizes the need for coordination between macroscopic, submicroscopic, and symbolic domains.^128,129^ Our findings add specificity by suggesting grade-level benchmarks based on teacher expectations, a topic underexplored in the literature.^130^ Compared to previous studies,^57,131^ which focused on undergraduates, our data offer insights into representational learning trajectories starting much earlier.

### Compounds Used to Introduce Representational Forms (RQ1.2)

Teachers often choose simple compounds such as ethanol, water, and methane to introduce structural representational forms. More complex compounds such as sulfur dioxide were used selectively to highlight rule exceptions (e.g., octet violations/expanded octets). This suggests that teachers choose examples that balance cognitive load and illustrative potential.^12^ Our results indicate that the chosen compounds align with textbook examples^26^ while also reflecting personal pedagogical experience and a preference for representational clarity.^132^ These results suggest a more teacher-driven approach and contrast earlier work,^30^ which categorized textbook examples by conceptual depth.

These observations suggest that these structures and molecules may be regarded as too complex to introduce new methods or to support translations between different representational forms. Consequently, teachers gravitate toward simpler and smaller structures. This aligns with pedagogical strategies that emphasize building foundational knowledge through gradually increasing complexity, thereby supporting the development of robust representational competence.^12,51,81,128^

### Common Student Errors When Producing Representational Forms (RQ2.1)

Across representational forms, teachers most frequently reported (i) missing or excess structural elements (e.g., atoms and bonds), (ii) problems with electron accounting (e.g., omitted/misplaced electron pairs and octet-rule violations), and (iii) misapplication of spatial conventions (e.g., wedge–dash notation).^26,133^ In addition, difficulties related to syntax and transfer between representational forms were repeatedly noted, especially for mixed and hybrid notations.

Teachers frequently reported that students rely on heuristic shortcuts (e.g., symmetry, analogy) rather than structural rules, echoing findings from previous studies.^57,134^ Interestingly, teachers also described domain-specific errors unique to hybrid and spatial representational forms, such as mixing conventions or ignoring stereochemical implications. These findings extend prior literature^6,12,128^ by highlighting hybrid and mixed representational forms as especially error-prone.^133^ This has received limited attention in previous cognitive research on chemical representational forms.

Teachers also noted that some students can produce formally correct structures in a largely procedural way, without using them as tools for chemical reasoning. In our data, this perspective is consistent with reports coded as inappropriate drawing practices and with frequent references to syntax/transfer difficulties, suggesting that correctness at the symbol level does not necessarily imply semantic understanding. This resonates with prior work showing that students may treat Lewis formulas as end products rather than as resources for explaining and predicting chemical behavior.^135,136^

### Compounds Used to Diagnose Common Errors (RQ2.2)

Teachers identified a core set of compounds whose representational features make them particularly effective for revealing misconceptions (e.g., dichloromethane for spatial understanding, SO_2_ for octet exceptions, and H_2_O for lone pairs). This supports prior claims^135^ that simple molecules can serve as “conceptual triggers” in diagnostic assessment. Such diagnostic choices appear to be driven more by the didactic clarity of representations than by chemical relevance, suggesting that representational salience is critical in compound selection. This observation supports the hypothesis that teachers prefer to demonstrate representational forms and the transfer between them using simpler structures. This approach aligns with pedagogical principles that emphasize building foundational knowledge through simpler molecules before transitioning to more complex structures, ensuring students develop a solid conceptual understanding.

### Strategies to Address Common Errors (RQ3)

Teachers described a range of instructional strategies, including repetition, scaffolded exercises, and error-based learning.^130,137^ Many emphasized the importance of using incorrect examples to stimulate metacognitive reflection—a strategy also advocated in the literature.^138^ Exercises were considered most effective when conceptually focused (e.g., one error per exercise), and repetition was seen as key to transitioning from rule-based reasoning to flexible representational fluency. Some interviewees discussed learning paths that gradually increase in abstractness, moving from ball-and-stick models to skeletal formulas via hybrid forms.^9^

The interviewees did not explicitly mention “individualized learning paths”. However, their emphasis on a structured and scaffolded progression suggests that curricula and teaching materials could benefit from offering such guidance. From a pedagogical perspective, this supports the idea of representational progressions that are aligned with concept difficulty and visual abstraction. Such progressions could also include diagnostic tasks with deliberate errors, helping students to deepen their conceptual understanding through active reflection and correction.

## Limitations

While this study offers valuable insights, several limitations must be acknowledged. First, the survey and interview samples were modest in size and drawn from a specific region. Participating teachers were volunteers and may not reflect the broader population of chemistry educators. As such, the findings—particularly the proportion of teachers endorsing certain views—should not be overgeneralized. Second, our data capture enacted practice from teacher reports. Where standards fully specify representational forms and timing, a curriculum-only analysis may suffice; however, for notations not explicitly named in the standards, teacher reports are necessary to make enacted choices visible. Hence, the data relied on self-reported teacher perceptions. Survey responses and interview narratives are inherently subjective and may be influenced by personal teaching experiences, recall biases, or social desirability effects. For instance, teachers might attribute difficulties to students rather than reflecting on their own instructional challenges. Without supporting data from student assessments or classroom observations, we cannot definitively verify that all the difficulties described reflect actual student struggles—though they are consistent with patterns reported in the literature.

The survey instrument, while carefully designed, was not previously validated. Some questions may have been interpreted inconsistently across respondents. Although efforts were made to clarify terminology, such variation may have influenced response patterns. Moreover, the limited use of open-ended survey items restricted the depth of explanation available in quantitative responses, making the interviews essential for elaboration. This again underscores the impact of the small qualitative sample on the study’s interpretive breadth. A further limitation concerns the lack of direct triangulation between teacher perceptions and student data. No classroom observations or student work samples were collected, and as a result, we cannot confirm whether the teacher-reported difficulties manifest in actual student performance. Additionally, all interviews followed the survey, which could have shaped the interviewees’ focus—a potential instrumentation bias. Although we used a semistructured and open-ended protocol to mitigate this, some influence from the survey cannot be ruled out. Moreover, although no significant differences in questionnaire responses were found between interviewees and noninterviewees, the possibility of a positive selection cannot be entirely ruled out.

Because participants had previously seen a list of example compounds (RQ1.1), a mild priming effect on the subsequent open-ended questions on compounds used to introduce representational forms (RQ1.2) cannot be ruled out. We mitigated this by using a broad compound list, inserting an intervening projections block to create temporal separation, and employing open text responses. Notably, RQ1.2 answers included many compounds not in the earlier list and converged with interview findings, suggesting that any priming, if present, was minimal.

The educational context also limits generalizability. Teachers operated within a particular national curriculum, using specific textbooks and representation conventions. Therefore, some reported difficulties—such as those with skeletal formulas—may be more pronounced in systems with similar instructional sequences. In contexts where different representational forms or teaching strategies are emphasized, student difficulties may differ in nature or frequency. Furthermore, although many teacher examples referred to organic compounds (e.g., alkanes), the study addressed school chemistry more broadly, including both organic and inorganic topics. The findings may therefore not generalize to all subfields of chemistry equally, such as physical chemistry, where different representational challenges arise.

Finally, our interpretations of underlying causes for student difficulties—such as cognitive overload or reliance on flawed heuristics—are inferred from teacher narratives and prior literature. While plausible, these interpretations remain hypothetical in the absence of direct student interviews or think-aloud protocols. For example, the suggestion that students rely on symmetry as a drawing heuristic is supported by teacher observation but not directly validated with student data.

## Implications

### Implications for Further Research

First, there is a need to compare teachers’ perceptions of student difficulties with *actual* student performance data. While our study captured what teachers *think* students struggle with, future studies could directly assess students’ abilities to read and draw structures (e.g., through diagnostic tests or think-aloud problem-solving sessions) and see how well this aligns with teacher expectations. Such research can validate teachers’ intuitions or reveal gaps. For instance, if teachers overlooked certain systematic errors students make (or vice versa), that would be important for refining pedagogical focus. Further research should design and test instructional interventions aimed at improving representational competence in chemistry. This could include experimental studies where one group of students is taught using enhanced strategies (for example, an explicit curriculum on how to interpret and draw various chemical representational forms, or integrating molecular modeling kits or digital visualization tools), and then comparing their outcomes with a control group. Prior studies have shown that prompting students to draw can enhance learning,^13,139^ but we need more evidence on how to best support students in drawing and reading chemical structures specifically. Interventions using molecular models, animations, and AR/VR environments have shown promise in improving students’ conceptual understanding and spatial reasoning.^140^ Future research should investigate which supports are most effective for helping students read and produce chemical representations. Research questions might include: *What teaching strategies most effectively reduce cognitive load during structure drawing?* and *Can students be trained to recognize and avoid common misconceptions, such as assuming molecular symmetry inappropriately?* While the added value of AR for fostering an intuitive grasp of spatial features was highlighted, the potential cognitive load from translating between 2D and 3D should be further investigated.^90,141^ Beyond this, future research should address two further issues. First, measurement approaches could be refined to improve reliability: for instance, by using multiple examples for a given representational form to ensure that responses refer to the category rather than to a single illustrative case. Second, while the present study focused on how and when teachers employ specific representational forms, future studies should also investigate *why* they choose certain forms in particular contexts (for a conceptual framework for capturing these underlying rationales and thus advancing our understanding of teachers’ representational practices, see SI, Section 4).

### Implications for Chemistry Teaching

Our findings on teachers’ reports of typical student errors and difficulties provide several implications for chemistry instruction. In particular, recurring errors and difficulties such as omitted atoms, incomplete valences, and incorrect spatial orientation suggest that instructional strategies need to address the conventions of chemical representational forms, rather than assuming that students will infer them. For instance, unpacking skeletal formulas—explaining that vertices represent carbon atoms and unshown hydrogens complete valences—may help prevent early misconceptions.^142^ Simple classroom activities like jointly reading a structure can build a mental checklist for students and demystify common representational forms. Several interviewees confirmed that dedicating time to this improved comprehension, echoing Kozma et al. on the value of making representational practices explicit.^5,12^

Teachers in our study also highlighted that difficulties arise when students are confronted with different representational forms. This finding underscores the importance of representational flexibility in instruction. Instructors should present molecules using multiple representational forms—Lewis, skeletal, ball-and-stick, physical, or digital—and encourage students to switch between them. Asking students to translate between representational forms (e.g., from 3D model to Lewis formula) helps build flexible representational thinking and addresses diverse learning preferences.^139,143^ Such integration fosters a deeper, more connected understanding of molecular structure. Rather than teaching structure drawing as a sequence of steps, instruction should emphasize the “why” behind these steps. Tying procedures to principles such as the octet rule and formal charge provides context for drawing choices.

The study further showed that many teachers view errors as opportunities for learning, especially when students are asked to critique or improve flawed structures. This aligns with prior research highlighting the value of addressing misconceptions directly. Teachers should actively confront common misconceptions—for instance, clarifying that molecules are not necessarily symmetrical. Presenting flawed example drawings for critique can help students recognize and correct errors. Such strategies normalize misunderstanding as a step in learning and help students let go of faulty heuristics.

Finally, our results indicate that teachers emphasize gradual increases in representational complexity and the need for repeated practice. This aligns with the established didactic progression in chemistry education. Structure drawing should be introduced with scaffolded support, starting with partial structures or prompts, then gradually increasing complexity. Checkpoints during drawing (e.g., after assigning frameworks or lone pairs) allow early error detection. Process-oriented instruction—guiding how students draw, not just what—was also highlighted by teachers as effective, and prior studies indicate that this approach builds competence and confidence more effectively than outcome-based feedback alone.^139,143,144^

Because difficulties with syntax and transfer were reported across multiple representational forms, instruction may benefit from explicitly practicing *translation* between notations (e.g., Lewis ↔ valence-line ↔ skeletal) alongside isolated practice within each notation. Translation tasks can enable students to cross-validate structural features (atoms, bonds, electron pairs, spatial conventions) across representations, which may help reduce recurring errors and strengthen representational fluency.

### Implications for the Design of Supporting Multimedia Tools

The difficulties reported by teachers—such as omission of atoms, incorrect spatial orientation, and challenges in translating between representations—point to areas where educational technology could provide targeted support. Prior research has shown that multimedia tools, including molecular models, animations, and interactive 2D/3D environments, can foster representational competence and spatial reasoning.^139,144,145^ Building on these findings, future tools might focus on dynamic visualization, error recognition, and adaptive feedback to address the specific errors highlighted by teachers in this study.

Students frequently apply structural rules procedurally, without deep understanding. Interactive software could guide students through the drawing process and provide immediate, context-sensitive feedback. For instance, an application could flag a carbon atom with five bonds or a missing formal charge, explaining the issue in real time. Existing tools like *ChemDraw* or *MarvinSketch* support advanced users, but simplified, gamified tools could serve beginners. Embedding common misconceptions into the system’s logic—for example, prompting students to justify a structure beyond surface heuristics—may make feedback more pedagogically effective.

Many difficulties stem from interpreting 2D structures in 3D space. Tools that dynamically link 2D drawings with 3D models (e.g., ball-and-stick or space-filling) can help bridge this gap.^86,145^ Augmented Reality (AR), which was also mentioned by interviewees, could extend this approach by allowing students to view and manipulate a 3D model in real space, thereby supporting transitions between 2D and 3D representations.^34,83,89,90^

Software can also be used to diagnose student misconceptions. If a student repeatedly omits lone pairs or misuses stereochemistry, an intelligent tutor could detect these patterns and assign targeted practice. Class-wide analytics can help teachers identify which representational concepts need reinforcement, while also providing researchers with process-level data to refine both software and pedagogy.

### Implications for Teacher Preparation

Effective chemistry instruction requires a combination of strong content knowledge, pedagogical strategies, and awareness of common misconceptions.^142,146^ Teacher education should ensure that pre-service teachers master all major representational forms—Lewis formulas, skeletal formulas, and 3D models—and can fluently translate between them. This includes hands-on drawing exercises and reflection on their own reasoning. Even high school teachers benefit from deeper exposure to advanced topics like stereochemistry or isomerism, as a solid conceptual foundation enhances their explanations of simpler concepts. Programs should focus on pedagogical content knowledge^146^ specific to representations—how to introduce them, sequence learning tasks, and address common errors. Case-based training using student misconceptions (e.g., incorrect Lewis formulas) helps teachers develop targeted interventions and feedback strategies. Sharing scaffolded tasks and analogy-based explanations from experienced educators fosters a community of best practices. New teachers must recognize typical student errors—e.g., overreliance on symmetry or rigid interpretations of the octet rule—and learn how to surface and address them. Training should include formative assessments that probe student thinking and encourage a reflective, inquiry-driven teaching stance. Given the value of multimedia tools, teachers need training not only in their technical use but also in pedagogically effective implementation. Learning to evaluate and integrate apps, drawing tools, and visualizations during training increases the likelihood of meaningful classroom use.

## Conclusion

The study highlights several key findings regarding teachers’ perceptions of the teaching and learning of chemical structural representational forms. According to their reports, simpler representational forms such as sum and condensed formulas are expected to be mastered before progressing to more complex representational forms, including those with spatial information such as wedge–dash or skeletal formulas. Teachers generally reported that students are usually expected to read these representational forms before they are required to draw them. In terms of chemical compounds, teachers reported frequently using alkanes, like methane and ethane, to introduce representational forms and diagnose typical errors. More complex organic compounds, such as amines or ketones, were rarely mentioned due to their perceived difficulty. Commonly reported student errors include omission or excess of atoms, violations of the octet rule, incorrect spatial orientation, and difficulties with syntax and transfer. The prevalence of these errors varies according to the specific representational form employed.

It is important to note that these findings reflect teachers’ expectations and classroom practices—the enacted curriculum—rather than direct evidence of students’ actual performance. Our results therefore provide insights into how teachers conceptualize representational learning and how they structure progression in the classroom, but they do not measure students’ achieved competence. This distinction is crucial for interpreting the data and for situating our findings within the broader context of chemistry education research. Complementary to our teacher-focused perspective, empirical studies have assessed representational competence as a construct distinct from general content knowledge, with recent contributions providing further evidence.^51,147^ Such research offers an important counterpart to our findings, as it captures student performance while our study documents teachers’ expectations and enacted practices.

Overall, the findings emphasize a pedagogical preference for starting with simple structures and gradually introducing complexity to build representational competence while minimizing cognitive load. Repetition and practice, organized by difficulty and conceptual focus, are seen as essential for reducing errors and improving understanding. Despite these instructional strategies, the findings also highlight the need for innovative tools to support students in overcoming challenges and correcting misconceptions in the long term. Digital applications, particularly those incorporating interactive and visual elements, could play an important role here. Features such as dynamic 3D visualization, error recognition, and adaptive feedback tailored to typical student errors could provide targeted support and individualized learning paths. Such tools would not only address current limitations in teaching practice but also enable students to progress at their own pace, ultimately fostering deeper understanding and greater success in chemistry education.

## Supplementary Material



## Notes

Extended background on terminology, compact taxonomy of the representational forms used in this study and a glossary of abbreviations, overviews of explicit curricular references to structural representations, inventory of how representations appear in textbooks, questionnaire materials, complete results tables, Teachers’ Representation Acceptance Model (TRAM), and additional context on the OrChemSTAR project (PDF)
