# Supplementary material for: Chemistry Teachers’ Perception of Students’ Difficulties in Reading and Drawing Chemical Structures
Source: J Chem Educ. 2026 Feb 24;103(4):1723–42. doi: 10.1021/acs.jchemed.5c00204 (PMC13085249; doi:10.1021/acs.jchemed.5c00204)
Supplement: Supplementary file 1 [file ed5c00204_si_001.pdf]

Supporting Information: Chemistry Teachers' Perception of Students' Difficulties in Reading and Drawing Chemical Structures

Lars-Jochen Thoms,*,†,‡ Gina Blick,†,‡ Lukas Schmidt,‡ Florian Furrer,¶ Mitra Purandare,§ Frieder Loch,§ and Johannes Huwer†,‡

†*Chair of Science Education, Thurgau University of Teacher Education, Kreuzlingen 8280, Switzerland*

‡*Chair of Science Education, Department of Chemistry, University of Konstanz, Konstanz 78464, Germany*

¶*Thurgau University of Teacher Education, Kreuzlingen 8280, Switzerland*

§*Department of Computer Science, Eastern Switzerland University of Applied Sciences, Rapperswil 8640, Switzerland*

E-mail: lars.thoms@phtg.ch

About this file

This Supporting Information (SI) contains extended background, terminology, curriculum and textbook overviews, instrument texts, and complete results tables referenced in the main article. Items in this SI are cited in the main text as "SI, Table S1", "SI, Figure S1", etc.

1 Terminology and Abbreviations

Several terms relevant to this study are used somewhat interchangeably in the literature—e.g., "formula," "structure," "notation," "representation," and "representational form." To guide readers, we include a compact taxonomy of the representational forms used in this study (Figure S1). The diagram distinguishes forms by the degree of structural and spatial information conveyed—ranging from molecular formulas (no structural information), through low-structure formulas (limited structural information), to structural formulas (e.g., Lewis, Kekulé, skeletal) and spatial representations (e.g., wedge–dash). Within the latter, projection formulas (Mills, Fischer, Haworth, Newman, Sawhorse) are shown as a dedicated subclass. This hierarchy clarifies our use of *representational forms* (a class defined by shared conventions) versus *representations* (a specific instance for a given compound and task). Table S1 provides a glossary of the abbreviations used in the figures and throughout the manuscript and SI.

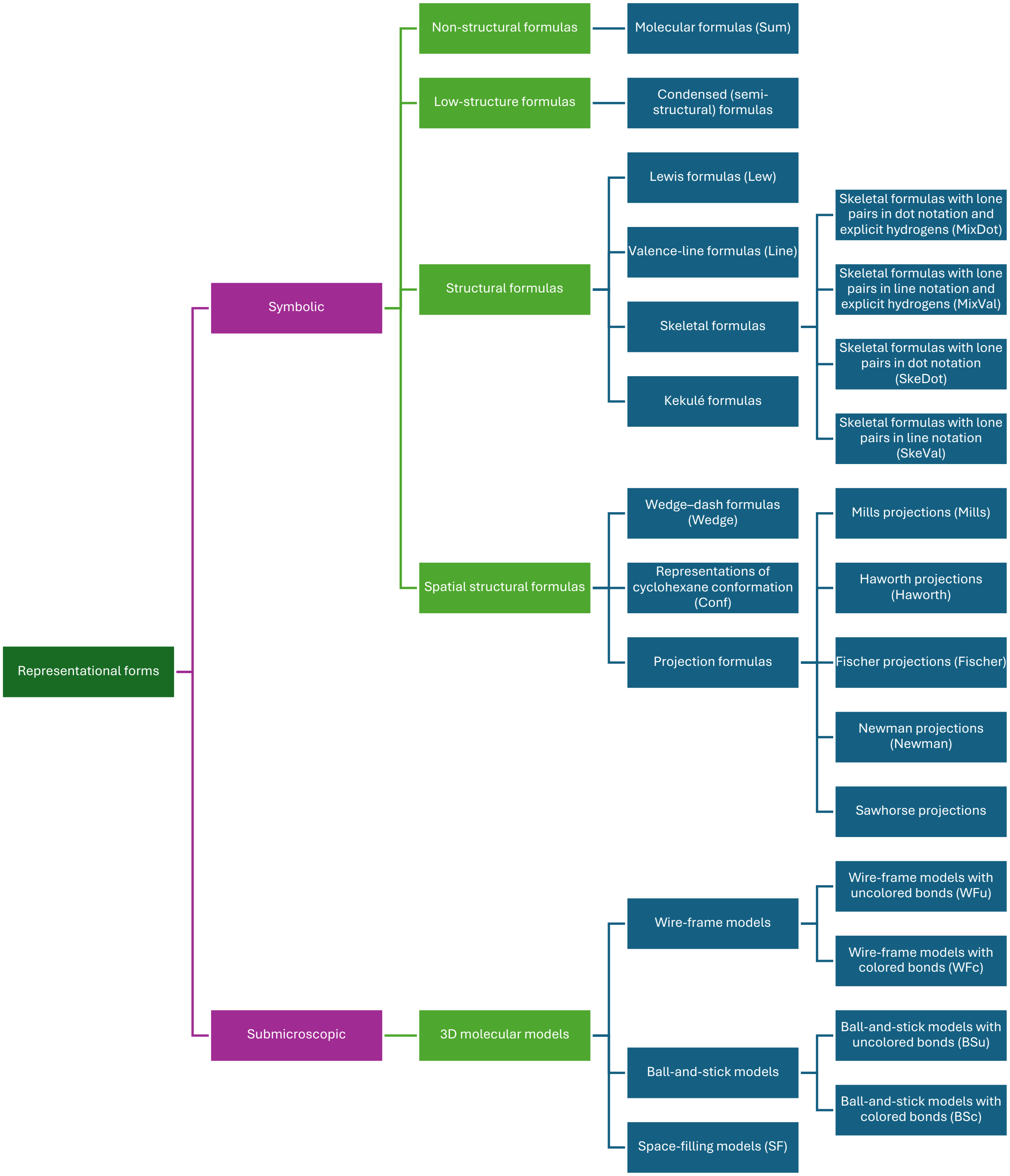

Figure S1: Hierarchy of representational forms used in this study. Abbreviations in parentheses match those used throughout the manuscript and SI.

Table S1: Glossary of abbreviations for representational forms used throughout the manuscript and Supporting Information.

Abbreviation	Representational forms
BS	Ball and stick models
BSc	Ball and stick models with colored bonds
BSu	Ball and stick models with uncolored bonds
Che	Chemical formulas
Cond	Condensed (semni-strucutral) formulas
Fischer	Fischer projections
Gas	Electron gas model
Gen	Generic mentions of molecular representations
Haworth	Haworth projections
Lew	Lewis formulas
Line	Valence-line formulas
Mills	Mills projections
MixDot	Skeletal formulas with lone pairs in dot notation and explicit hydrogens
MixVal	Skeletal formulas with lone pairs in line notation and explicit hydrogens
Newman	Newman projections (Newman)
Orb	Orbital models
SF	Space filling models
SkeDot	Skeletal formulas with lone pairs in dot notation
Skel	Skeletal formulas
SkeVal	Skeletal formulas with lone pairs in line notation
Sum	Molecular formulas
WFc	Wire frame models with colored bonds
WFu	Wire frame models with uncolored bonds

2 Explicit References to Specific Structural Representational Forms in Curricula

The ability to express chemical relationships using appropriate notations is emphasized as a core competency in national science education standards or curricula, such as the U.S. Next Generation Science Standards[S1], the Swiss framework curriculum for Matura schools of the Swiss Conference of Cantonal Ministers of Education[S2], the Danish national curricula[S3], the German national education standards[S4], and German state curricula, for example, in Baden–Württemberg[S5].

The degree of specificity and the extent to which forms of molecular representation are mentioned in curricula vary considerably both across countries and among states or cantons within a country. Table S2 provides an overview, distinguishing between mentions that are either generic or specify only broad families of molecular representations—namely chemical formulas (Che), Lewis formulas (Lew, often without clarifying whether electron-dot or valence-line notation is intended), orbital model representations (Orb), and fully generic mentions of molecular representations (Gen)—and explicit references to particular methods, such as skeletal formulas (Skel), the electron gas model (Gas), condensed (semi-structural) formulas (Cond), valence-line formulas (Line), wedge–dash formulas (Wedge), specific projections such as Haworth (Haworth) and Fischer (Fischer), and three-dimensional models such as ball-and-stick (BS) and space-filling (SF). Mixed structural representations, chair/boat conformations, Mills and Newman projections, and wireframe models were not mentioned in any of the curricula we screened (Tab. S2).

In Germany, some federal states provide very detailed and comprehensive descriptions of structural representations (e.g., Berlin–Brandenburg[S6], Bremen[S7,S8], Rhineland-Palatinate[S9], Saarland[S10,S11], and Thuringia[S12]), whereas others limit themselves to more generic requirements (e.g., Mecklenburg–Western Pomerania[S13,S14], North Rhine–Westphalia[S15,S16]). There are also differences with regard to projection formulas: Bavaria[S17], Bremen[S7,S8],

Rhineland–Palatinate[S9], Saxony[S18], Schleswig–Holstein[S19], and Thuringia[S12] explicitly require knowledge of Haworth and Fischer projections, while Hamburg[S20,S21], Lower Saxony[S22], Mecklenburg–Western Pomerania[S13,S14], North Rhine–Westphalia[S15,S16], and Saarland[S10,S11,S23,S24] do not. Three-dimensional molecular representations are predominantly required only in a generic sense; among the curricula analyzed, only two explicitly included the space-filling model (SF)[S10,S13].

The Swiss national framework curriculum for secondary schools is strongly competence-oriented and provides no specific guidance on the forms of representation to be taught. Accordingly, cantonal curricula vary considerably: some specify several explicit representation forms (e.g., Aargau[S25] and Solothurn[S26]), whereas most remain unspecific, leaving the choice to teachers. Requirements for knowledge of the space-filling model (SF) are relatively frequent[S25–S31].

In the United States, the curricula and examination standards examined provide little guidance on modes of molecular representation. Where three-dimensional representations are mentioned, the ball-and-stick model (BS) predominates[S32–S36].

Table S2: Structural representations explicitly named in curricula. AT: Austria, CH: Switzerland, DE: Germany, US: United States.

Nat.	State	Level	ISCED	Source	Che	Lew	Orb	Gen	Skel	Gas	Cond	Line	Wedge	Haworth	Fischer	BS	SF	Ref.
AT	Vienna	Gymnasium	3	AHS Oberstufe - Lehrplan für das UF Chemie (2016)			✓											S37
CH	Aargau	Gymnasium	3	Neue Kantonsschule Aarau Fachlehrpläne		✓	✓		✓	✓			✓				✓	S25
CH	Appenzell-Innerhoden	Gymansium	3	NA														S38
CH	Basel-Country	Volksschule	2	Lehrplan mit Stoffinhalten, Themen und Treffpunkten Chemie.													✓	S27
CH	Basel-Stadt	Gymnasium	3	NA				✓										S39
CH	Bern	Gymnasium	3	Lehrplan 17 für den gymnasialen Bildungsgang						✓								S40
CH	Fribourg	Gymnasium	3	Lehrplan der Gymnasialstudien Studienbereich Naturwissenschaften Chemie														S41
CH	Geneva	Gymnasium	3	Plan d'études				✓										S42
CH	Glarus	Gymnasium	3	Lehrplan Gymnasium Oberstufe		✓	✓											S43
CH	Graubünden	Gymnasium	3	Biologie und Chemie														S44
CH	Lucerne	Gymnasium	3	Lehrpläne MAR-Klassen - Kantonsschule Sursee													✓	S28
CH	Lucerne	Gymnasium	3	Kantonsschule Reussbühl Luzern, Lehrplan 2021			✓										✓	S29
CH	Neuchâtel	Gymnasium	3	NA														S45
CH	Obwalden	Gymansium	3	Biologie / Chemie Schwerpunktfach Biologie / Chemie			✓											S46
CH	Obwalden	Gymnasium	3	Chemie Grundlagenfach Chemie				✓										S47
CH	Schaffhausen	Volksschule	2	Natur, Mensch, Gesellschaft														S48
CH	Solothurn	Gymnasium	3	NA		✓	✓		✓	✓			✓				✓	S26

continued on next page

Nat.	State	Level	ISCED	Source	Che	Lew	Orb	Gen	Skel	Gas	Cond	Line	Wedge	Haworth	Fischer	BS	SF	Ref.
CH	St. Gallen	Gymnasium	3	Lehrplan für das Gymnasium im Kanton St.Gallen		✓												S49
CH	Thurgau	Gymnasium	3	Maturitätsplan Thurgau			✓											S50
CH	Valais	Gymnasium	3	Lehrplan der Walliser Fachmittelschule														S51
CH	Vaud	Gymnasiun	3	Ecole de maturité														S52
CH	Zug	Gymnasium	3	Lehrplan Chemie für das Grundlagenfach		✓											✓	S30
CH	Zug	Gymnasium	3	Biologie und Chemie (Chemischer Teil) Lehrplan für das Schwerpunktfach		✓	✓	✓									✓	S31
CH	Zurich	Gymansium	3	Kantonsschule Stadelhofen Zürich Stundentafel und Lehrplan Mathematisch-naturwissenschaftliches Profil mit Schwerpunktfach Biologie und Chemie			✓											S53
DE	Baden-Württemberg	Gymansium	3	Chemie - Überarbeitete Fassung vom 25. März 2022		✓	✓							✓	✓			S5
DE	Bavaria	Gymnasium	3	LehrplanPLUS - Chemie 12 (erhöhtes Anforderungsniveau)			✓											S54
DE	Bavaria	Gymnasium	3	LehrplanPLUS - Chemie 11 (NTG)				✓						✓	✓			S17
DE	Berlin and Brandenburg	Gymnasium	3	Rahmenlehrplan für die gymnasiale Oberstufe Teil C Chemie					✓		✓		✓		✓			S6
DE	Brandenburg	Gymansium	2	Rahmenlehrplan für die Sekundarstufe I Jahrgangsstufen 7 – 10				✓										S55
DE	Brandenburg	Sec. 1	2	Rahmenlehrplan für die Sekundarstufe I Jahrgangsstufe 7-10 Hauptschule Realschule Gesamtschule Gymnasium Chemie														S56
DE	Bremen	Gymnasium	3	Lehrplan Chemie		✓	✓					✓		✓	✓			S8
DE	Bremen	Gymnasium	3	Chemie Bildungsplan für die Gymnasiale Oberstufe – Einführungsphase und Qualifikationsphase		✓	✓			✓			✓	✓	✓			S7
DE	Hamburg	Gymnasium	2	Bildungsplan Gymnasium Sekundarstufe I Chemie	✓	✓												S20
DE	Hamburg	Gymnasium	3	Bildungsplan Studienstufe Chemie														S21

continued on next page

Nat.	State	Level	ISCED	Source	Che	Lew	Orb	Gen	Skel	Gas	Cond	Line	Wedge	Haworth	Fischer	BS	SF	Ref.
DE	Lower Saxony	Sec. 1 + 2	3	Kerncurriculum für das Gymnasium – gymnasiale Oberstufe die Gesamtschule – gymnasiale Oberstufe das Berufliche Gymnasium das Abendgymnasium das Kolleg		✓			✓		✓							S22
DE	Mecklenburg-Western Pomerania	Gymnasium	2.3	Rahmenplan für die Sekundarstufe I Gymnasium, Gesamtschule		✓		✓									✓	S13
DE	Mecklenburg-Western Pomerania	Gymnasium	3	Rahmenplan für die Sekundarstufe 2														S14
DE	North Rhine-Westphalia	Gymansium	2	Kernlehrplan für das Gymnasium – Sekundarstufe I in Nordrhein-Westfalen Chemie														S15
DE	North Rhine-Westphalia	Gymnasium	3	Kernlehrplan für die Sekundarstufe II Gymnasium/Gesamtschule in Nordrhein-Westfalen Chemie														S16
DE	Rhineland-Palatinate	Gymnasium	2.3	Lehrplan Chemie			✓		✓	✓				✓	✓			S9
DE	Saarland	Gymnasium	3	Chemie Lehrplan Naturwissenschaftlicher Zweig Gymnasiale Oberstufe Einführungsphase			✓											S23
DE	Saarland	Gymnasium	3	Chemie Lehrplan Sprachlicher Zweig Gymnasiale Oberstufe Einführungsphase			✓											S24
DE	Saarland	Gymnasium	3	Chemie Lehrplan Gymnasiale Oberstufe Leistungskurs Redaktionell veränderte Fassung (Juli 2024)		✓			✓	✓		✓					✓	S10
DE	Saarland	Gymnasium	3	Chemie Lehrplan Gymnasiale Oberstufe Grundkurs Redaktionell veränderte Fassung (Juli 2024)					✓	✓	✓	✓						S11
DE	Saxony	Gymnasium	2.3	Lehrplan Gymnasium Chemie			✓			✓				✓	✓			S18
DE	Schleswig-Holstein	Sec. 1 + 2	2.3	Fachanforderungen Chemie			✓							✓	✓			S19
DE	Thuringia	Gymansium	2.3	Thüringer Lehrplan für den Erwerb der allgemeinen Hochschulreife Chemie 2024		✓	✓		✓	✓	✓	✓		✓	✓			S12
US	Alaska	High School	3	K-12 Science Standards for Alaska														S57

continued on next page

Nat.	State	Level	ISCED	Source	Che	Lew	Orb	Gen	Skel	Gas	Cond	Line	Wedge	Haworth	Fischer	BS	SF	Ref.
US	Arizona	High School	3	Science High School Curriculum Guides including Standards, Three Dimensional Foundations, and Evidence of Learning Specifications Board Approval February 2020				✓										S58
US	Arkansas	High School	3	Chemistry II		✓	✓											S59
US	NA	College, Admission Examination	3	AP Chemistry, Course and Exam Description	✓	✓	✓									✓	✓	S60
US	Colorado	High School	3	Colorado Academic Standards Science				✓										S61
US	Conneticut	High School	3	Core Science Curriculum Framework An Invitation for Students and Teachers to Explore Science and Its Role in Society			✓											S62
US	Delaware	High School	3	Unit Topical Arrangement				✓										S63
US	District of Columbia	High School	3	District of Columbia Science Pre-K through Grade 12 Standards	✓			✓										S64
US	Florida	High School	3	Florida's State Academic Standards for Science	✓													S65
US	Georgia	High School	3	Science Georgia Standards of Excellence														S66
US	Hawaii	High School	3	Next Generation Science Standards * Performance Expectations Arranged By Disciplinary Core Idea (DCI)			✓											S67
US	Idaho	High School	3	High School Chemistry Essential Standards Extended Guide		✓										✓		S32
US	Illinois	High School	3	Illinois State Board of Education-State Course Catalog	✓													S68
US	Indiana	High School	3	Science and Engineering Process Standards (SEPS)	✓			✓										S69
US	Iowa	High School	3	Iowa Academic Standards for Science														S70
US	Kansas	High School	3	NA														S71

continued on next page

Nat.	State	Level	ISCED	Source	Che	Lew	Orb	Gen	Skel	Gas	Cond	Line	Wedge	Haworth	Fischer	BS	SF	Ref.
US	Kentucky	High School	3	Kentucky Science Standards					✓									S72
US	Lousiana	High School	3	Lousiana Student Science														S73
US	Maine	High School	3	Maine Science And Engineering Standards				✓										S74
US	Maryland	High School	3	NA														S75
US	Massachussetts	High School	3	Science and Technology/Engineering Learning Standards	✓	✓		✓										S76
US	Michigan	High School	3	Michigan K-12 Standards Science November 2015														S77
US	Minnesota	High School	3	Minnesota K-12 Academic Standards in Science Education 2019 Adopted Version, effective September 27, 2021 Spreadsheet Version Introduction														S78
US	Mississippi	High School	3	Suggested Instructional Planning Guide For The Mississippi College- And Career-Readiness Standards		✓		✓								✓		S33
US	Missouri	High School	3	6-12 Science Grade-Level Expectations Missouri Department of Elementary and Secondary Education Spring 2016				✓										S79
US	Montana	High School	3	Montana Science Content Standards														S80
US	Nebraska	High School	3	Nebraska's College And Career Ready Standards For Science														S81
US	Nevada	High School	3	Nevada Science Standards														S82
US	New Hampshire	High School	3	DCI Arrangements of the Next Generation Science Standards														S83
US	New Jersey	High School	3	Chemistry-Performance Expectations by Unit														S84
US	New Mexico	High School	3	NM STEM Ready! Science Standards New Mexico Specific Standards														S85
US	New York	High School	3	New York State P-12 Science Learning Standards			✓	✓										S86
US	North Carolina	High School	3	North Carolina Standard Course Of Study K-12 Science, Chemistry				✓										S87

continued on next page

Nat.	State	Level	ISCED	Source	Che	Lew	Orb	Gen	Skel	Gas	Cond	Line	Wedge	Haworth	Fischer	BS	SF	Ref.
US	North Dakota	High School	3	North Dakota Science Content Standards Grades K-12 February 2019				✓										S88
US	Ohio	High School	3	Ohio's Learning Standards and Model Curriculum	✓	✓										✓		S34
US	Oklahoma	High School	3	Oklahoma Academic Standards for Science	✓													S89
US	Oregon	High School	3	2022 Oregon Science Standards	✓													S90
US	Pennsylvania	High School	3	Science, Technology and Engineering, and Environmental Literacy and Sustainability Standards (STEELS)														S91
US	Rhode Isalnds	High School	3	NA														S92
US	South Carolina	High School	3	Chemistry Performance Targets	✓	✓										✓		S35
US	South Dakota	High School	3	South Dakota Science Standards														S93
US	Tennessee	High School	3	Tennessee Academic Standards for Science		✓	✓											S94
US	Texas	High School	3	Chapter 112. Texas Essential Knowledge and Skills for Science	✓	✓		✓										S95
US	Utah	High School	3	Utah Science With Engineering Education (Seed) Standards		✓										✓		S36
US	Virginia	High School	3	Science Standards of Learning for Virginia Public Schools	✓		✓											S96
US	Washington	High School	3	NA														S97
US	Wisconsin	High School	3	Wisconsin Standards For Science														S98
US	Wyoming	High School	3	2023 Science (Emended Feb. 2025) Wyoming Content and Performance Standards (WYCPS)														S99
US	NA	High School	3	High School Physical Sciences														S100

3 Occurrence of Structural Representations in Chemistry Textbooks

Table S3: Structural representations in chemistry textbooks. DE–BW: Baden–Württemberg, Germany; DE: Germany, US: United States, UK: United Kingdom.

Book title	State	Sum	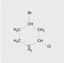 Line	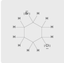 MixDot	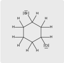 MixVal	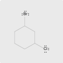 LinDot	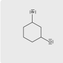 LinVal	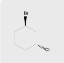 Wedge	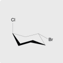 Conf	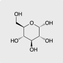 Mills	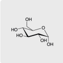 Conf	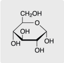 Haworth	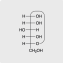 Fischer	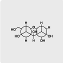 Newman	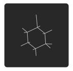 WFu	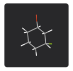 WFc	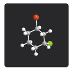 BSu	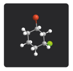 BSc	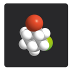 SF	Ref.
Elemente Chemie	DE-BW	10	19	222	31		133	174				181	190				19	350	18	S101
Elemente Chemie, Klasse 8-10	DE-BW	124	223	221	221		223	223	332								220		220	S102
Chemie 1	DE-BW	61																		S103
Chemie 2	DE-BW	14	127	127	128												131			S104
Chemie 3	DE-BW	15	16	16	16												73		85	S105
Elemente Chemie II	DE-BW	16	16		287	45	285		211		346	376	345				16		117	S106
Organic Chemistry	UK	12	143		180	112	214	2	1	127	3		316	364			18			S107
Chemie. Das Basiswissen der Chemie.	DE	127	128	120				502	500		564	564	564	500			141			S108
Fachwerk Chemie	DE-BW	96	127	127	128							323					275		97	S109
Chemie Gesamtband 11-12	DE-BW	15	22	22	22				191		147	187	146				22		153	S110
Chemie heute, Sek II	DE	16	16		287	45	285		211		346	376	345				16		117	S111
PubChem	US							Yes								Yes		Yes	Yes	S112
Wikipedia (Eng), 2023	US	Yes	Yes	Yes	No	No	No	Yes	Yes	No	Yes	Yes	Yes	Yes	No	No	No	No	No	S113
Wikipedia (Eng), 2024	US	Yes	Yes	Yes	No	No	No	Yes	Yes	No	Yes	Yes	Yes	Yes	No	No	No	No	No	S114
Wikipedia (Eng), 2025	US	Yes	Yes	Yes	No	No	No	Yes	Yes	No	Yes	Yes	Yes	Yes	No	No	No	No	No	S115

4 Conceptual Model of Teachers' Representation Acceptance (TRAM)

A large fraction of what ultimately appears in chemistry classrooms is shaped not only by the *intended* curriculum (national or state frameworks, school-level syllabi) but by the *enacted* curriculum—the content and materials that teachers actually choose to emphasize and assess[S116]. This distinction is of particular significance for representational forms not explicitly mandated in official documents or only mentioned in passing (for example, referring to "projections" without naming specific types like Fischer or Haworth), since their use in class then depends largely on teachers' choices[S117]. In such cases, the classroom presence is unlikely to be determined by curricula alone; in practice, it hinges on individual instructors' priorities and comfort with those forms[S118]. Even when a particular representation is explicitly named in the curriculum (e.g., Lewis structures), its classroom implementation is mediated by teachers' professional judgment and a persistent adherence to prior content norms[S116], as well as by the availability and framing of textbooks and school materials—well-known filters between policy and practice[S119]. In our context, textbooks exert a particularly strong influence on teachers' representational choices and on how consistently conventions are introduced and scaffolded, which can in turn amplify or dampen the presence of specific notations in daily instruction[S119–S121]. This aligns with prior research showing that the representations provided (and sometimes inconsistently presented) in textbooks shape teachers' planning and students' representational experiences[S121–S123], often introducing mixed or hybrid forms that confuse learners and lead to documented misunderstandings[S119].

To capture these enactment dynamics and to justify a teacher survey, we treat each specific representation (e.g., Lewis structures, wedge–dash stereochemical drawings, Fischer or Haworth projections) as a pedagogical "technology" in the broad sense—an intentional tool for mediating learning. The central acceptance mechanism is therefore described by an extended Technology Acceptance Model (TAM)[S124,S125] chain adapted to representations

(Fig. S2).

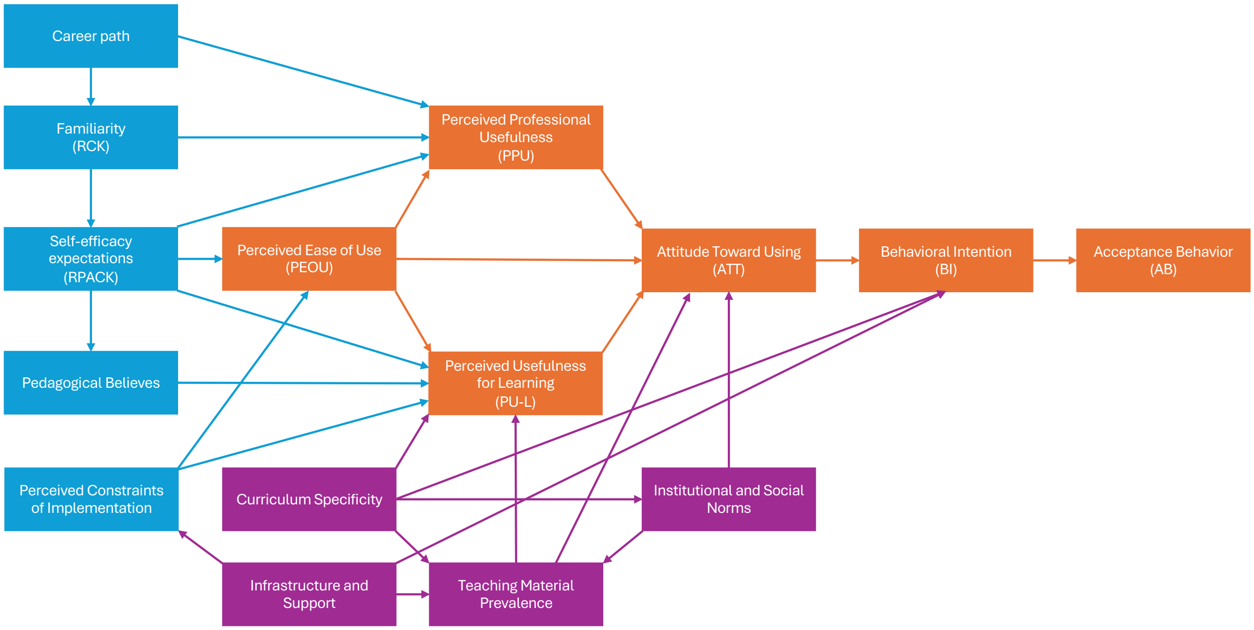

Figure S2: Teachers' Representation Acceptance Model (TRAM). Orange shows the core TAM chain (PEOU → ATU → BI → AB); blue shows teacher-related determinants (RPACK/self-efficacy, familiarity, beliefs); purple shows contextual determinants (curriculum specificity, teaching-material prevalence, institutional norms, support/constraints). PEOU also increases usefulness judgments for learning (PU-L) and professional preparation (PPU). The TRAM is conceptual and used solely to justify the teacher survey; no path coefficients are estimated.

Prior work in chemistry/STEM contexts supports the expected sequence from perceived ease of use (PEOU) to attitude (ATT), to behavioral intention (BI), and, finally, to actual classroom use (acceptance behavior, AB). In a large study with secondary chemistry teachers[S126], PEOU and PU-L positively predicted ATT, ATT predicted BI, and BI predicted AB. Similar directions were observed in recent secondary studies of digital/visual representation tools[S127,S128], with qualitative work also noting an intention–use gap[S129]. The usefulness–attitude link is additionally reinforced by a chemistry meta-analysis and a systematic review[S130,S131]. Moreover, PEOU has been shown to feed into PU-L (and into perceived professional usefulness, PPU), and both PU-L and PPU contribute to ATT[S126].

The teacher-centered determinants for adoption are described by the Technological Pedagogical and Content Knowledge (TPACK)[S132] framework: selecting and using a notation

productively requires content knowledge about what the representation expresses, pedagogical knowledge about when and how to introduce it, and technological/representational knowledge about its syntax, semantics, and typical student pitfalls. In our model, this representation-specific professional self-efficacy expectations are summarized as RPACK (Representational Pedagogical and Content Knowledge), which is expected to increase perceived ease of use.

Representation-specific knowledge and self-efficacy (RPACK/TPACK-consistent) are associated with higher PEOU and more favorable attitudes toward using representations[S123,S133–S135]. Familiarity (prior use/exposure) increases PEOU and sometimes directly influences ATT[S126]; it is also shaped by disciplinary background and career path, which can privilege certain representational practices[S117]. Long-standing findings that pedagogical beliefs guide choices of models/representations further explain variance in ATT beyond ease and usefulness[S122,S136–S138].

Where curricula explicitly specify notations, teachers tend to perceive those representations as more instructionally useful (PU-L), and they are more likely to plan or intend to use them[S119,S120,S122,S138–S140]. Conversely, when a representation is not named in standards, the curriculum cannot be the primary driver of its classroom presence, shifting explanatory weight to teacher-related factors. Teaching-material prevalence (textbooks, exam exemplars) raises familiarity and can reduce perceived constraints[S119,S121]. Institutional support (e.g., PD, time, resources) reliably eases implementation (Support→PEOU)[S120,S123,S127,S141], whereas perceived constraints (syllabus pressure, assessment alignment, workload) depress intention and/or use (Constraints→BI/AB)[S126,S128]. Social/departmental norms can also steer intentions[S123].

Because many target representations in our study (e.g., stereochemical projections beyond canonical Lewis structures) are not systematically mandated, understanding *when* teachers expect students to interpret versus generate them, and *why* they select them for instruction, requires asking teachers directly. TRAM therefore serves here as a map of

plausible determinants of use—organizing teacher-related and contextual influences around the core acceptance chain—while the present paper uses teacher survey and interviews to describe current practice rather than to statistically test this model.

5 The OrChemSTAR Project

This study is situated within the broader OrChemSTAR program (Organic Chemistry Science Teaching and Learning with Augmented Reality), a multi-phase effort to support students in learning to read and draw structural formulas and to translate between two- and three-dimensional representations of chemical structures[S142,S143]. In OrChemSTAR, a mobile iOS application is being developed and evaluated with a learning mode (camera-based capture of student drawings with automated error feedback and adaptive practice) and an AR mode (overlay of 3D molecular models on instructional materials)[S144–S146]. The present manuscript does not report tool development; rather, it provides the empirical groundwork needed to make such a tool instructionally relevant and acceptable in Swiss upper-secondary (Matura) schools. Specifically, our survey and interviews were designed to (i) identify which representational forms teachers *actually* employ in class, (ii) determine *when* teachers expect students to interpret versus generate each form, (iii) compile commonly used introductory compounds and typical student errors, and (iv) document teacher strategies for supporting students. Eliciting these practitioner judgments allowed us to align OrChemSTAR's content scope, error taxonomy, and example library with prevalent Swiss classroom practice, rather than with idealized assumptions. A second, pragmatic aim was early teacher engagement: Chemistry teachers might act as gatekeepers and multipliers for innovations at school and cantonal levels; consulting them at an early stage could increase the likelihood of later uptake and inform realistic implementation constraints. In keeping with participatory/teacher-informed design traditions in chemistry education, we therefore use a mixed-methods approach (national survey plus follow-up interviews) to connect teachers' ra-

tionales and observed difficulties to concrete design requirements and evaluation criteria for OrChemSTAR[S142–S146]. Further background on the project's objectives, target population, and planned evaluations is available on the project website[S142,S146].

6 Questionnaire Materials

Table S4: Welcome text informing about the questionnaire's aims.

In chemistry lessons, many different ways of representing chemical structures are used (e.g., structural formulas). However, students must first learn to read and create these representations themselves in order to understand the technical concepts.
In the Swiss National Science Foundation-funded project "OrChemSTAR - Organic Chemistry Science Teaching and Learning with Augmented Reality," we are investigating the difficulties students have in reading and writing chemical structures and developing an app to support the learning process.
However, we need your help!
By answering a few questions about the use of structural formulas in your chemistry lessons and your experiences with student learning, you will help us find the best possible examples for learning formula notation.

Thank you very much for supporting our research!

7 Complete Results Tables

Table S5: Frequently used chemical compounds to introduce different notations. Mentions per main category (in italics) and per term category. The maximum of five most frequently mentioned term categories per main category are shown.

Compound	Sum	Line	MixVal	LinVal	Wedge	Conf	MixDot	LinDot	Total
Alkanes	*28*	*26*	*8*	*11*	*1*	*11*	*16*	*13*	*114*
Methane	20	7	4	6	0	0	12	2	51
Alkanes	3	8	1	3	0	5	2	4	26
Ethane	2	4	2	2	0	1	2	1	14
Propane	2	3	0	0	1	2	0	4	12
Hexane	1	4	1	0	0	3	0	2	11
Alcohols	*15*	*13*	*3*	*11*	*6*	*11*	*3*	*5*	*67*
Ethanol	14	9	3	9	0	6	1	4	46
Alcohols	0	2	1	1	0	4	0	1	9
Methanol	1	1	1	1	0	1	0	1	6
Propanol	0	1	0	0	0	2	0	0	3
Isopropanol	0	0	0	0	0	1	0	0	1

Compound	Sum	Line	MixVal	LinVal	Wedge	Conf	MixDot	LinDot	Total
Inorganic compounds	*14*	*3*	*9*	*11*	*4*	*1*	*0*	*0*	*42*
Oxygen	10	0	4	2	1	0	0	0	17
Hydrogen	3	0	2	5	1	1	0	0	12
Nitrogen	1	2	2	2	1	0	0	0	8
Fluorine	1	0	1	2	1	0	0	0	5
Iodine	0	0	0	0	0	0	0	0	0
Sugars	*12*	*1*	*0*	*0*	*0*	*1*	*2*	*21*	*37*
Glucose	10	0	1	1	0	1	0	12	25
Sugars	2	0	1	0	0	0	0	4	7
Fructose	0	0	0	0	0	0	0	4	4
Maltose	0	0	0	0	0	0	0	1	1
Saccharose	0	0	0	0	0	0	0	0	0
Carboxylic acids	*3*	*7*	*4*	*6*	*1*	*3*	*1*	*1*	*26*
Acetic acid	3	6	4	5	1	2	1	1	23
Carboxylic Acids	0	1	1	1	0	3	0	0	6
Acetylsalicylic acid	0	0	0	0	0	0	0	0	0
Glyoxylic acid	0	0	0	0	0	0	0	0	0
Cycloalkanes	*1*	*7*	*2*	*0*	*1*	*5*	*2*	*6*	*24*
Cyclohexane	1	6	2	0	0	1	2	7	19
Cycloalkanes	0	1	0	0	1	4	0	0	6
Cyclopropane	0	0	0	0	0	0	0	0	0
Haloalkanes	*2*	*1*	*4*	*2*	*1*	*7*	*2*	*3*	*22*
Haloalkanes	1	1	0	0	0	7	0	4	13
Bromomethane	0	0	0	2	0	0	2	1	5
Bromopropane	1	0	0	0	0	0	0	0	1
Dibromopropane	0	0	0	0	1	0	0	0	1
Bromohexane	0	0	0	0	0	0	0	0	0
Aldehydes	*2*	*1*	*0*	*3*	*0*	*1*	*0*	*1*	*8*
Methanal	1	1	0	3	0	1	0	0	6
Ethanal	1	0	0	0	0	0	0	1	2
Propanal	0	0	0	0	0	0	0	0	0
Alkenes	*0*	*1*	*0*	*0*	*0*	*4*	*3*	*4*	*12*
Alkenes	0	1	0	0	0	2	1	2	6
Propene	0	0	0	0	0	1	0	2	3
Pentene	0	0	0	0	0	1	0	1	2
Ethene	0	0	0	0	0	0	0	1	1
Propadiene	0	0	0	0	0	0	0	0	0
Aromatic compounds	*0*	*2*	*0*	*0*	*0*	*1*	*1*	*1*	*5*
Benzene	0	2	0	0	0	1	0	2	5
Phenol	0	0	0	0	0	0	0	0	0
Ethers	*1*	*1*	*1*	*1*	*0*	*1*	*0*	*0*	*5*
Dimethyl ether	1	1	1	1	0	1	0	0	4
Diethyl ether	0	0	0	0	0	0	0	0	0
Halocycloalkanes	*1*	*1*	*0*	*0*	*0*	*1*	*0*	*0*	*3*
Bromocyclohexane	1	0	0	0	0	1	0	1	3
2-Dibromocyclohexane	0	0	0	0	0	0	0	0	0
Alkynes	*0*	*1*	*0*	*0*	*0*	*3*	*0*	*1*	*5*
Alkynes	0	1	0	0	0	1	0	0	2
Propyne	0	0	0	0	0	1	0	1	2
Ethyne	0	0	0	0	0	1	0	0	1
Alkaloids	*2*	*0*	*0*	*0*	*1*	*0*	*0*	*0*	*3*
Nicotine	1	0	0	0	1	0	0	0	2
Caffeine	1	0	0	0	0	0	0	0	1
Ketones	*0*	*0*	*1*	*0*	*0*	*1*	*0*	*0*	*2*

Compound	Sum	Line	MixVal	LinVal	Wedge	Conf	MixDot	LinDot	Total
Acetone	0	0	1	0	0	1	0	0	2
Organic compounds	*0*	*1*	*0*	*0*	*0*	*0*	*0*	*1*	*2*
Indigo dye	0	1	0	0	0	0	0	1	2
Hydrocarbons	*0*	*0*	*0*	*0*	*0*	*0*	*0*	*0*	*0*
Hydrocarbons	0	0	0	0	0	0	0	0	0
Carbohydrates	*0*	*0*	*0*	*0*	*0*	*0*	*0*	*0*	*0*
Carbohydrates	0	0	0	0	0	0	0	0	0
Elements	*0*	*0*	*0*	*0*	*0*	*0*	*0*	*0*	*0*
Carbon	0	0	0	0	0	0	0	0	0

Table S6: Frequently used chemical compounds to diagnose common mistakes by notation.

Compound	Sum	Line	MixVal	LinVal	Wedge	Conf	MixDot	LinDot	Total
Alkanes	*9*	*7*	*2*	*2*	*0*	*2*	*6*	*6*	*34*
Methane	3	1	0	0	1	0	2	2	9
Alkanes	3	2	2	0	0	2	2	0	9
Ethane	3	1	0	1	0	0	1	1	7
Hexane	2	0	0	1	0	0	0	0	4
Propane	1	0	0	0	1	0	1	0	3
Alcohols	*7*	*7*	*3*	*6*	*3*	*4*	*2*	*2*	*34*
Ethanol	6	2	0	1	0	0	2	5	16
Propanol	1	0	0	0	0	0	1	5	7
Cyclohexanole	1	0	0	0	0	0	1	4	6
Hexanole	1	0	0	0	0	0	1	1	3
Isopropanol	1	0	0	0	0	0	1	0	2
Carboxylic acids	*5*	*5*	*2*	*5*	*1*	*5*	*3*	*1*	*27*
Acetic acid	4	4	2	5	1	5	3	1	25
Carboxylic Acids	1	1	0	0	0	0	0	0	2
Acetylsalicylic acid	0	0	0	0	0	0	0	0	0
Glyoxylic acid	0	0	0	0	0	0	0	0	0
Sugars	*0*	*1*	*0*	*0*	*0*	*0*	*3*	*14*	*18*
Glucose	0	0	0	0	0	0	3	9	12
Fructose	0	0	0	0	0	0	0	3	3
Maltose	0	0	0	0	0	0	0	2	2
Saccharose	0	0	0	0	0	0	0	1	1
Sugars	0	0	0	0	0	0	0	0	0
Alkenes	*2*	*4*	*2*	*3*	*0*	*1*	*3*	*1*	*16*
Ethene	2	0	0	0	0	0	2	2	6
Alkenes	2	1	0	0	0	0	1	1	5
Propene	1	0	0	0	0	0	1	3	5
Pentene	0	0	0	0	0	0	0	0	0
Propadiene	0	0	0	0	0	0	0	0	0
Inorganic compounds	*6*	*0*	*0*	*2*	*0*	*0*	*0*	*0*	*8*
Hydrogen	4	0	0	0	0	0	0	0	4
Oxygen	2	0	0	0	0	0	0	0	2
Nitrogen	1	0	0	0	0	0	0	0	1
Fluorine	1	0	0	0	0	0	0	0	1
Iodine	0	0	0	0	0	0	0	0	0
Cycloalkanes	*1*	*2*	*2*	*1*	*0*	*2*	*0*	*1*	*9*
Cyclohexane	1	0	0	0	0	0	1	2	4
Cycloalkanes	1	2	0	0	0	2	0	0	4

Compound	Sum	Line	MixVal	LinVal	Wedge	Conf	MixDot	LinDot	Total
Cyclopropane	1	0	0	0	0	0	0	0	1
Haloalkanes	*0*	*0*	*0*	*2*	*1*	*3*	*0*	*1*	*7*
Haloalkanes	0	0	0	0	0	3	1	1	5
Bromomethane	0	0	0	0	0	0	0	1	1
Bromopropane	0	0	0	0	0	0	0	1	1
Dichloromethane	0	0	0	0	0	0	0	0	0
Bromochlorofluoroiodomethane	0	0	0	0	0	0	0	0	0
Aldehydes	*2*	*0*	*0*	*2*	*0*	*1*	*0*	*0*	*5*
Methanal	2	0	0	0	0	0	1	1	4
Propanal	0	0	0	0	0	0	1	0	1
Ethanal	0	0	0	0	0	0	0	0	0
Alkynes	*1*	*1*	*1*	*0*	*0*	*0*	*1*	*1*	*5*
Propyne	0	0	0	0	0	0	1	2	3
Ethyne	1	1	0	0	0	0	0	0	2
Ethers	*1*	*0*	*1*	*1*	*0*	*2*	*0*	*0*	*5*
Diethyl ether	1	0	0	1	0	0	1	0	3
Dimethyl ether	0	0	1	0	0	0	1	0	2
Aromatic compounds	*0*	*2*	*0*	*0*	*1*	*0*	*0*	*0*	*3*
Benzene	0	0	0	0	0	0	0	3	3
Phenol	0	0	0	0	0	0	0	0	0
Ketones	*0*	*0*	*0*	*1*	*0*	*2*	*0*	*0*	*3*
Acetone	0	0	0	0	0	0	1	2	3
Halocycloalkanes	*2*	*0*	*0*	*0*	*0*	*0*	*1*	*0*	*3*
Bromocyclohexane	1	0	0	0	0	0	1	0	2
2-Dibromocyclohexane	1	0	0	0	0	0	0	0	1
Alkaloids	*2*	*0*	*0*	*0*	*0*	*0*	*0*	*0*	*2*
Caffeine	1	0	0	0	0	0	0	0	1
Nicotine	1	0	0	0	0	0	0	0	1

References

(S1) National Research Council *Next generation science standards: For states, by states*; The National Academies Press: Washington, DC, 2013.

(S2) Schweizerische Konferenz der kantonalen Erziehungsdirektoren (EDK) *Rahmenlehrplan für die Maturitätsschulen*; EDK: Bern, Switzerland, 1994.

(S3) Børne- og Undervisningsministeriet *Vejledning Kemi A, B, C - stx*; Børne- og Undervisningsministeriet: København, 2024.

(S4) Kultusministerkonferenz *Bildungsstandards im Fach Chemie für den Mittleren Schulabschluss*; Kultusministerkonferenz, 2004.

(S5) Ministerium für Kultus, Jugend und Sport Bildungsplan des Gymnasiums Chemie. 2022.

(S6) Landesinstitut für Schule und Medien Berlin-Brandenburg (LISUM) Rahmenlehrplan Für Die Gymnasiale Oberstufe. Teil C. Chemie. 2022.

(S7) Die Senatorin für Kinder und Bildung, Freie Hansestadt Bremen Chemie. Bildungsplan für die Gymnasiale Oberstufe. Einführungsphase und Qualifikationsphase. 2010.

(S8) Curriculumsentwicklung. Landesinstitut für Schule. Abteilung 2 Chemie. Bildungsplan für die Gymnasiale Oberstufe. Einführungsphase und Qualifikationsphase. 2022.

(S9) Rheinland-Pfalz Ministerium für Bildung Lehrplan Chemie. Grund- und Leistungsfach in der gymnasialen Oberstufe (Mainzer Studienstufe). 2022.

(S10) Ministerium für Bildung und Kultur Saarland Chemie Lehrplan Gymnasiale Oberstufe Leistungskurs Redaktionell Veränderte Fassung (Juli 2024). 2024.

(S11) Ministerium für Bildung und Kultur Saarland Chemie. Lehrplan Gymnasiale Oberstufe, Grundkurs, Redaktionell veränderte Fassung (Juli 2024). 2023.

(S12) Thüringer Ministerium für Bildung, Jugend und Sport Lehrplan Chemie, Gymnasium (2024). 2024.

(S13) Ministerium für Bildung und Kindertagesförderung, Institut für Qualitätsentwicklung Mecklenburg-Vorpommern Rahmenplan Für Die Qualifikationsphase Der Gymnasialen Oberstufe, Chemie, 2022, Erprobungsfassung. 2022.

(S14) Ministerium für Bildung und Kindertagesförderung, Institut für Qualitätsentwicklung Mecklenburg-Vorpommern Rahmenplan Für Den Sekundarbereich II. Fachgymnaisium/Abendgymnasium. Chemie. 2022, Erprobungsfassung. 2022.

(S15) Ministerium für Schule und Bildung des Landes Nordrhein-Westfalen Kernlehrplan für die Sekundarstufe I Gymnasium in Nordrhein-Westfalen, Chemie. 2019.

(S16) Ministerium für Schule und Weiterbildung des Landes Nordrhein-Westfalen Kernlehrplan Für Die Sekundarstufe II, Gymnasium/Gesamtschule in Nordrhein-Westfalen. Chemie. 2014.

(S17) Staatsinstitut für Schulqualität und Bildungsforschung LehrplanPLUS – Chemie 11.

(S18) Staatsministerium für Kultus, Freistaat Sachsen Lehrplan Gymnasium. Chemie. 2004/2007/2009/2011/2019/2022/2025. 2025.

(S19) Schleswig-Holstein Ministerium für Bildung, Wissenschaft und Kultur Fachanforderungen Chemie. Allgemein Bildende Schulen, Sekundarstufe I, Sekundarstufe II. 2022.

(S20) Freie und Hansestadt Hamburg Behörde für Schule und Berufsbildung Bildungsplan Gymnasium, Sekundarstufe I, Chemie. 2024.

(S21) Freie und Hansestadt Hamburg Behörde für Schule und Berufsbildung Bildungsplan Studienstufe Chemie. 2022.

(S22) Niedersächsisches Kultusminsterium Kerncurriculum für das Gymnasium - gymnasiale Oberstufe, die Gesamtschule - gymnasiale Oberstufe, das Berufliche Gymnasium, das Abendgymnasium, das Kolleg. Chemie. 2017.

(S23) Ministerium für Bildung und Kultur Saarland Chemie. Lehrplan Naturwissenschaftlicher Zweig, Gymnasiale Oberstufe, Einführungsphase. 2024.

(S24) Ministerium für Bildung und Kultur Saarland Chemie. Lehrplan Sprachlicher Zweig, Gymnasiale Oberstufe, Einführungsphase. 2024.

(S25) Neue Kantonsschule Aarau Neue Kantonsschule Aarau, Fachlehrpläne. 2013.

(S26) Amt für Berufsbildung, Mittel- und Hochschulen, Solothurn Lehrplan Gymnasium. Kantonsschule Olten.,Kantonsschule Solothurn. 2014.

(S27) Bildungs-, Kultur- und Sportdirektion Lehrplan Mit Stoffinhalten, Themen Und Treffpunkten Chemie. 2021.

(S28) Dienststelle Gymnasialbildung Kanton Luzern Lehrpläne MAR-Klassen Kantonsschule Sursee. 2021.

(S29) Kantonsschule Reussbühl Luzern Chemie. 2021.

(S30) Kantonsschule Zug | Gymnasium Chemie. Lehrplan Für Das Grundlagenfach. 2018.

(S31) Kantonsschule Zug | Gymnasium Biologie und Chemie (Chemischer Teil). Lehrplan für das Schwerpunktfach. 2010.

(S32) Idaho Department of Education High School Chemistry. Essential Standards Extended Guide. High School Chemistry. 2014.

(S33) Mississippi Department of Education Suggested Instructional Planning Guide for the Mississippi College- and Career-Readiness Standards. Science. Chemistry. 2021.

(S34) Ohio Department of Education Ohio's Learning Standards and Model Curriculum Science. 2018.

(S35) Chemistry Instructional Unit Resource SCDE, Office of Standards and Learning South Carolina Academic Standards and Performance Indicators for Science 2014. 2014.

(S36) Utah State Board of Education Utah Science with Engineering Education (SEEd) Standards. 2023.

(S37) Bildungsdirektion Wien Chemie. 2016.

(S38) Gymnasium St. Antonius Appzenzell Lehrpläne Gymnasium St. Antonius, Appenzell. 2011.

(S39) Erziehungsdepartment des Kantons Basel-Stadt, Mittelschulen und Berufsbildung Lehrplan Gymnasium. 2018.

(S40) Erziehungsdirektion des Kantons Bern Lehrplan 17 Für Den Gymnasialen Bildungsgang. 2016.

(S41) Direction de l'instruction publique, de la culture et du sport. Direktion für Erziehung, Kultur und Sport. Canton de Fribourg /Kanton Freiburg. Lehrplan Der Gymnasialstudien. Studienbereich Naturwissenschaften, Chemie.

(S42) République et Canto de Genève, Départment de l'instruction publique, Enseignement secondaire II Plan D'Etudes. 2018.

(S43) Kantonsschule Glarus Lehrplan für das Gymnasium (3. bis 6. Klassen). 2019.

(S44) Bündner Kantonsschule Biologie Und Chemie.

(S45) Conférence suisse des directeurs cantonaux de l'instruction publique (CDIP) Plan d'études Cadre Pour Les Écoles de Maturité. 2024.

(S46) Kantonsschule Obwalden Sarnen. Bildungs- und Kulturdepartement, Amt für Volks- und Mittelschulen. Biologie / Chemie. 2021.

(S47) Kantonsschule Obwalden Sarnen. Bildungs- und Kulturdepartement, Amt für Volks- und Mittelschulen. Chemie. 2021.

(S48) Deutschschweizer Erziehungsdirektoren-Konferenz Lehrplan 21, Kanton Schaffhausen. Natur, Mensch, Gesellschaft. 2018.

(S49) Gymnasien des Kantons St.Gallen Lehrplan für das Gymnasium im Kanton St.Gallen. 2006.

(S50) Schulamt II Kanton Thurgau Lehrplan der thurgauischen Maturitätsschulen (LTM 96). 1996.

(S51) Service de l'enseignement SION Plan d'études Des Écoles de Culture Générale Du Valais (ECG). Lehrplan Der WAlliser Fachmittelschulen (FMS). 2018.

(S52) Département de l'enseignement et de la formation professionnelle, Direction générale de l'enseignement postobligature, Canton de Vaud Ecole de maturité. Plan d'études et liste des examens. 2024.

(S53) Bildungsrat des Kantons Zürich Kantonsschule Stadelhofen Zürich. Stundentafel und Lehrplan. Mathematisch-naturwissenschaftliches Profil mit Schwerpunktfach Biologie und Chemie. 2011.

(S54) Staatsinstitut für Schulqualität und Bildungsforschung LehrplanPLUS – Chemie 12.

(S55) Langgymnasium und Fachmittelschule Winterthur, Kantonnschule Rychenberg Lehrplan Chemie.

(S56) Berlin Senatsverwaltung für Bildung, Jugend und Sport Rahmenlehrplan für die Sekundarstufe I. Jahrgangsstufe 7-10, Hauptschule, Realschule, Gesamtschule, Gymnasium. Chemie. 2006.

(S57) Alaska Department of Education and Early Development K-12 Science Standards for Alaska.

(S58) MESA Public Schools Arizona Science High School Curriculum Guides Including Standards, Three Dimensinoal Foundations, and Evidence of Learning Specifications. 2020.

(S59) Arkansas Department of Education Arkansas. K-12 Science Standards. Education for a New Generation. Chemistry II. 2016.

(S60) College Board AP Chemistry Course and Exam Description. College Board: New York, NY, 2024; © 2024 College Board.

(S61) Colorado Department of Education Colorado Academic Standards Science. High School. 2020.

(S62) State Department of Education Connecticut Core Science Curriculum Framework. An Invitation for Students and Teachers to Explore Science and Its Role in Society.

(S63) Delaware Department of Education Unit Topical Arrangement. 2025.

(S64) District of Columbia Public Schools. Office of Academic Services. District of Columbia. Science. Pre-K through Grade 12 Standards.

(S65) CPALMS. Browse and Search Standards. https://www.cpalms.org/public/search/Standard.

(S66) Georgia Department of Education Science Georgia Standards of Excellence. 2016.

(S67) Hawai'I State Department of Education Performance Expectations Arragned by Disciplinary Core Idea (DCI). 2014.

(S68) Illinois State Board of Education Illinois State Board of Education, State Course Catalog, SY 2025. 2025.

(S69) Indiana Department of Education Indiana Content Standards for Educators. Science-Chemistry. 2016.

(S70) Iowa Department of Education Iowa Academic Standards for Science. 2025.

(S71) Kansas State Department of Education Kansas Science Standards, High School Chemistry, Unpacked Standards. 2025.

(S72) Kentucky Department of Education Kentucky Department of Education - Course Standards. 2022.

(S73) Louisiana Department of Education Louisiana Student Standards Sciences, Luisiana Connectors, Chemistry.

(S74) Maine Department of Education Maine Science and Engineering Standards. 2019.

(S75) Maryland State Department of Education Science Branch. https://marylandpublicschools.org/about/Pages/DCAA/Science/index.aspx.

(S76) Massachusetts Department of Education Science and Technology/Engineering Learning Standards. 2016.

(S77) Michigan Department of Education Michigan K-12 Standards. Science. 2015.

(S78) Minnesota Department of Education Minnesota K-12 Academic Standards in Science Education. 2019 Adopted Version, Effective September 27, 2021. Spreadsheet Version. 2021.

(S79) Missouri Department of Elementary and Secondary Education 6-12 Science Grade Level Expectations. 2016.

(S80) Montana Board of Public Education Montana Science Content Standards. 2016.

(S81) Nebraska State Board of Education Nebraska's College and Career Ready Standards for Science. 2017.

(S82) Nevada Department of Education Nevada Science Standards.

(S83) Next Generation Science Standards. For sTates, By States DCI Arrangements of the Next Generation Science Standards. 2017.

(S84) State of New Jersey Department of Education Grades 9-12 Model Curriculum Framework. https://www.nj.gov/education/standards/science/Sci9-12.shtml.

(S85) NMPED Public Education Department NM STEM Ready! Science Standards New Mexico Specific Standards. 2018.

(S86) New York State Education Department New York State P-12 Science Learning Standards.

(S87) North Carolina State Board of Education Department of Public Instruction North Carolina Standard Course of Study. K-12 Science, Chemistry. 2023.

(S88) North Dakota Department of Public Instruction North Dakota Science Content Standards. Grades K-12. 2019.

(S89) Oklahoma Education Oklahoma Academic Standards Science. 2020.

(S90) Oregon Department of Education 2022 Oregon Science Standards. K-12 Science Education. 2022.

(S91) Pennsylvania Department of Education Science, Technology & Engineering, and Environmental Literacy & Sustainability Standards (STEELS). K-12 Standards. 2022.

(S92) Division of Teaching and Learning, Rhode Island Department of Education Selecting and Implementing a High-Quality Curriculum in RI. A Guidance Document. 2020.

(S93) South Dakota Department of Education South Dakota Science Standards. 2024.

(S94) Tennessee Department of Education (TDOE) Tennessee Academic Standards for Science. 2025.

(S95) Texas Education Agency (TEA) Chapter 112. Texas Essential Knowledge and Skills for Science. Subchapter C. High School. 2024.

(S96) Virginia Department of Education 2018 Virginia Science Standards of Learning Curriculum Framework. 2018.

(S97) Next Generation Science Standards DCI Arrangements of the Next Generation Science Standards. 2017.

(S98) Wisconsin Department of Public Instruction Wisconsin Standards for Science. 2017.

(S99) Wyoming Department of Education (WDE) Wyoming Content & Performance Standards (WYCPS). 2024.

(S100) Next Generation Science Standards. For States, By States DCI Arrangements of the Next Generation Science Standards. 2017.

(S101) Bee, U.; Blauth, O.; Gietz, P.; Maier, H.; Wiese, K. *Elemente Chemie Kursstufe. Schulbuch*, 1st ed.; Ernst Klett Verlag: Stuttgart Leipzig, 2022.

(S102) *Elemente Chemie 8-10. Schülerbuch*, 1st ed.; Ernst Klett Verlag.

(S103) Bohrmann-Linde, C. *Chemie Baden-Württemberg. 1, Schülerbuch: Schülerbuch / bearbeitet von Claudia Bohrmann-Linde [und weiteren]*, 1st ed.; C.C. Buchner.

(S104) Bohrmann-Linde, C. *Chemie Baden-Württemberg. 2, Schülerbuch: Schülerbuch / bearbeitet von Claudia Bohrmann-Linde [und weiteren]*; C.C. Buchner.

(S105) Bohrmann-Linde, C.; Colberg, F.; Faas, S.; Goltz, G.; Jäger, H.-J.; Kohn, T.; Krüger, J.; Schmitz, W.; Lässle, M.; Maucher, M.; Schneiderhan, K.; Hoffmann, A. In *Chemie Baden-Württemberg. 3 Schülerbuch: Schülerbuch 3 / bearbeitet vonClaudia Bohrmann-Linde, Frank Colberg, Stafanie Faas, Georg Goltz, Hans-Jürgen Jäger [und weitere]*; Bohrmann-Linde, C., Krüger, J., Schneiderhan, K., Eds.; Chemie Baden-Württemberg - neu; Buchner, C.C.

(S106) Eisner, W., Ed. *Elemente - Chemie: Unterrichtswerk. 2, Gesamtausg., [Schülerbd.]: Unterrichtswerk für die Sekundarstufe II / von Werner Eisner*, 1st ed.; Klett.

(S107) Clayden, J.; Greeves, N.; Warren, S. *Organic chemistry*, 2nd ed.; Oxford university press.

(S108) Mortimer, C. E.; Müller, U. *Chemie: Das Basiswissen der Chemie*, 13th ed.; Georg Thieme Verlag.

(S109) *Fachwerk Chemie. Schülerbuch*, 1st ed.; Cornelsen.

(S110) Bohrmann-Linde, C.; Siehr, I.; Eberhardt, S.; Englberger, R.; Faas, S.; Frings, A.; Goerz-Lorenz, B.; Heldt, N.; Hundt, K.; Jauernik, S.; Karus, C.; Kleefeldt, S.; Krüger, J.; Lässle, M.; Meuter, N.; Orth, J. M.; Schneiderhan, K.; Schönborn, A.; Tausch, M. *Chemie Baden-Württemberg Gesamtband 11-12. Schülerband*, 1st ed.; Buchner, C.C.

(S111) Förster, R.; Kallfelz, M.; Kaundinya, E. In *Chemie heute. Gesamtbd., [Schülerband] / herausgegeben von Ralf van Nek, Dr. Rolf Schulte-Coerne, Dr. Bernhard F. Sieve ; bearbeitet von Rosemarie Förster, Monika Kallfelz, Elisabeth Kaundinya [und 12 weiteren] ; Beratung Dr. Karl T. Risch*, [allgemeine ausgabe, gesamtschule, gymnasium], s II, druck a ed.; Nek, R. v., Schulte-Coerne, R., Sieve, B. F., Risch, K. T., Eds.; Westermann.

(S112) Kim, S.; Chen, J.; Cheng, T.; Gindulyte, A.; He, J.; He, S.; Li, Q.; Shoemaker, B.; Thiessen, P.; Yu, B.; Zaslavsky, L.; Zhang, J.; Bolton, E. PubChem 2025 update. *Nucleic Acids Research* **2025**, *53*, D1516–D1525.

(S113) Structural formula. `https://en.wikipedia.org/w/index.php?title=Structural_formula&oldid=1139837643`, Page Version ID: 1139837643.

(S114) Structural formula. `https://en.wikipedia.org/w/index.php?title=Structural_formula&oldid=1195121410`, Page Version ID: 1195121410.

(S115) Structural formula. `https://en.wikipedia.org/w/index.php?title=Structural_formula&oldid=1290079796`, Page Version ID: 1290079796.

(S116) Michael B. Burt; Sarah B. Boesdorfer The Implementation of Reform-Based Standards in High School Chemistry Classrooms Influenced by Science Teaching Orientations. *Electronic Journal for Research in Science & Mathematics Education* **2021**, *25*, 72–93.

(S117) Boesdorfer, S. B. Using Teachers' Choice of Representations to Understand the Translation of Their Orientation Toward Science Teaching to Their Practice. *Electronic Journal of Science Education* **2015**, *19*, 1–20.

(S118) Stowe, R. L.; Esselman, B. J. The Picture Is Not the Point: Toward Using Representations as Models for Making Sense of Phenomena. *Journal of Chemical Education* **2023**, *100*, 15–21.

(S119) Bergqvist, A.; Chang Rundgren, S.-N. The Influence of Textbooks on Teachers' Knowledge of Chemical Bonding Representations Relative to Students' Difficulties Understanding. *Research in Science & Technological Education* **2017**, *35*, 215–237.

(S120) Philipp, S. B.; Johnson, D. K.; Yezierski, E. J. Development of a Protocol to Evaluate the Use of Representations in Secondary Chemistry Instruction. *Chemistry Education Research and Practice* **2014**, *15*, 777–786.

(S121) Ferreira, J. E. V.; Lawrie, G. A. Profiling the Combinations of Multiple Representations Used in Large-Class Teaching: Pathways to Inclusive Practices. *Chemistry Education Research and Practice* **2019**, *20*, 902–923.

(S122) Bindernagel, J. A.; Eilks, I. Evaluating Roadmaps to Portray and Develop Chemistry Teachers' PCK about Curricular Structures Concerning Sub-Microscopic Models. *Chemistry Education Research and Practice* **2009**, *10*, 77–85.

(S123) Wu, M.-Y. M.; Yezierski, E. J. Investigating Teacher–Teacher Feedback: Uncovering Useful Socio-pedagogical Norms for Reform-Based Chemistry Instruction. *Journal of Chemical Education* **2023**, *100*, 4224–4236.

(S124) Davis, F. D. Perceived Usefulness, Perceived Ease of Use, and User Acceptance of Information Technology. *13*, 319.

(S125) Holden, H.; Rada, R. Understanding the Influence of Perceived Usability and Technology Self-Efficacy on Teachers' Technology Acceptance. *43*, 343–367.

(S126) Chroustová, K.; Šorgo, A.; Bílek, M.; Rusek, M. Differences in chemistry teachers' acceptance of educational software according to their user type: An application of extended UTAUT model. *Journal of Baltic Science Education* **2022**, *21*, 762–787.

(S127) Ripsam, M.; Nerdel, C. Teachers' Attitudes and Self-Efficacy toward Augmented Reality in Chemistry Education. *Frontiers in Education* **2024**, *8*.

(S128) Wohlfart, O.; Wagner, A. L.; Wagner, I. Digital Tools in Secondary Chemistry Education – Added Value or Modern Gimmicks? *Frontiers in Education* **2023**, *8*.

(S129) Popova, M.; Jones, T. Chemistry Instructors' Intentions toward Developing, Teaching, and Assessing Student Representational Competence Skills. *Chemistry Education Research and Practice* **2021**, *22*, 733–748.

(S130) Castro, R. The Effects of Chemistry Virtual Laboratories in Academic Achievement of Secondary Level Learners: A Meta-Analysis. *Integrated Science Education Journal* **2025**, *6*, 24–37.

(S131) Permatasari, M. B.; Rahayu, S.; Dasna, I. W. Chemistry Learning Using Multiple Representations: A Systematic Literature Review. *Journal of Science Learning* **2022**, *5*, 334–341.

(S132) Mishra, P.; Koehler, M. J. Technological Pedagogical Content Knowledge: A Framework for Teacher Knowledge. *Teachers College Record* **2006**, *108*, 1017–1054.

(S133) Khourey-Bowers, C.; Fenk, C. Influence of Constructivist Professional Development on Chemistry Content Knowledge and Scientific Model Development. *Journal of Science Teacher Education* **2009**, *20*, 437–457.

(S134) Tempel, B. J.; Randler, C.; Rehm, M.; Wilhelm, M. Model Comptences in Chemistry and Biology Lessons - What Skills Do Teachers Need? A Systematic Literature Review; Modellkompetenzen Im Chemie- Und Biologieunterricht - Welche Fähigkeiten Brauchen Lehrkräfte? Ein Systematisches Literaturreview. *Progress in Science Education (PriSE)* **2018**, Vol. 1 No. 1 (2018).

(S135) Can, H. B. Enacted Pedagogical Content Knowledge Profiles of Chemistry Teachers. *Journal of Educational Issues* **2021**, *7*, 565.

(S136) Bergqvist, A.; Drechsler, M.; Chang Rundgren, S.-N. Upper Secondary Teachers' Knowledge for Teaching Chemical Bonding Models. *International Journal of Science Education* **2016**, *38*, 298–318.

(S137) Wong, S.; Wong, A. S. L. In *E-Book Proceedings of the ESERA 2013 Conference: Science Education Research For Evidence-based Teaching and Coherence in Learning.*; Constantinou, C. P., Papadouris, N., Hadjigeorgiou, A., Eds.; European Science Education Research Association, 2014.

(S138) Eilks, I. *Innovations in Science Education and Technology*; Springer Netherlands, 2013; pp 213–230.

(S139) Georgiadou, A.; Tsaparlis, G. Chemistry teaching in lower secondary school with methods based on: a) psychological theories; b) the macro, representational, and submicro levels of chemistry. *Chemistry Education Research and Practice* **2000**, *1*, 217–226.

(S140) Schafer, A. G. L.; Borland, V. M.; Yezierski, E. J. Visualizing Chemistry Teachers' Enacted Assessment Design Practices to Better Understand Barriers to "Best Practices". *Chemistry Education Research and Practice* **2021**, *22*, 457–475.

(S141) Blasie, C.; Butler-Kahle, J. The Penn Science Teacher Institute: A Proven Model.

The Journal of Mathematics and Science: Collaborative Explorations **2009**, *11*, 41–55.

(S142) Thoms, L.-J.; Huwer, J. In *Progress in Digitalisation in Chemistry Education*; Huwer, J., Wilke, T., Banerji, A., Eds.; Waxmann: Münster, 2025; pp 113–118.

(S143) Thoms, L.-J.; Furrer, F.; Rhiner, M.; Bullock, M.; Rothlin, T.; Däullary, L.; Purandare, M.; Loch, F.; Huwer, J. In *Frühe Naturwissenschaftliche Bildung*; van Vorst, H., Ed.; Gesellschaft Für Didaktik Der Chemie Und Physik; GDCP, 2025; Vol. 44.

(S144) Purandare, M.; Rothlin, T.; Loch, F.; Huwer, J.; Thoms, L.-J. SMARE—Structure Matching and Recognition Engine for Hand-Drawn Chemical Formulas. Artificial Intelligence in Education. Cham, 2025; pp 124–132.

(S145) Däullary, L.; Loch, F.; Syskowski, S.; Huwer, J.; Thoms, L.-J. Evaluating Usability and User Experience of the OrChemSTAR Educational App Using Eye Tracking. Proceedings of the HCI International Conference 2025. 2025; In Press.

(S146) Thoms, L.-J. OrChemSTAR. 2025; `https://orchemstar.ch`, Accessed on February 26, 2025.
